# Exploring glutathione transferase and Cathepsin L-like proteinase for designing of epitopes-based vaccine against *Fasciola hepatica* by immunoinformatics and biophysics studies

**DOI:** 10.3389/fimmu.2024.1478107

**Published:** 2024-09-26

**Authors:** Hassan H. Alhassan, Muhammad Ikram Ullah, Abdurahman A. Niazy, Sami I. Alzarea, Omar Awad Alsaidan, Abdulaziz Ibrahim Alzarea, Aseel Awad Alsaidan, Abulaziz A. Alhassan, Muharib Alruwaili, Yasir S. Alruwaili

**Affiliations:** ^1^ Department of Clinical Laboratory Sciences, College of Applied Medical Sciences, Jouf University, Sakaka, Al-Jouf, Saudi Arabia; ^2^ Department of Oral Medicine and Diagnostic Sciences, College of Dentistry, King Saud University, Riyadh, Saudi Arabia; ^3^ Department of Pharmacology, College of Pharmacy, Jouf University, Sakaka, Al-Jouf, Saudi Arabia; ^4^ Department of Pharmaceutics, College of Pharmacy, Jouf University, Sakaka, Al-Jouf, Saudi Arabia; ^5^ Clinical Pharmacy Department, College of Pharmacy, Jouf University, Sakaka, Al-Jouf, Saudi Arabia; ^6^ Department of Family and Community Medicine, College of Medicine, Jouf University, Sakaka, Al-Jouf, Saudi Arabia; ^7^ Department of Pediatric, Domat Aljandal General Hospital, Ministry of Health, Domat Aljandal, Al-Jouf, Saudi Arabia; ^8^ Sustainable Development Research and Innovation Center, Deanship of Graduate Studies and Scientific Research, Jouf University, Sakaka, Saudi Arabia

**Keywords:** *Fasciola hepatica*, immunoinformatics, epitopes, molecular docking, molecular dynamic simulation analysis

## Abstract

Fasciolosis is a zoonotic infection and is considered a developing deserted tropical illness threatening ruminant productivity and causing financial losses. Herein, we applied immunoinformatics and biophysics studies to develop an epitopes vaccine against *Fasciola hepatica* using glutathione transferase and Cathepsin L-like proteinase as possible vaccine candidates. Using the selected proteins, B- and T-cell epitopes were predicted. After epitopes prediction, the epitopes were clarified over immunoinformatics screening, and only five epitopes, EFGRWQQEKCTIDLD, RRNIWEKNVKHIQEH, FKAKYLTEMSRASDI, TDMTFEEFKAKYLTE, and YTAVEGQCR were selected for vaccine construction; selected epitopes were linked with the help of a GPGPG linker and attached with an adjuvant through another linker, EAAAK linker. Cholera toxin B subunit was used as an adjuvant. The ExPASy ProtParam tool server predicted 234 amino acids, 25.86257 kDa molecular weight, 8.54 theoretical pI, 36.86 instability index, and −0.424 grand average of hydropathicity. Molecular docking analysis predicted that the vaccine could activate the immune system against *F. hepatica*. We calculated negative binding energy values. A biophysics study, likely molecular docking molecular dynamic simulation, further validated the docking results. In molecular dynamic simulation analysis, the top hit docked compounds with the lowest binding energy values were subjected to MD simulation; the simulation analysis showed that the vaccine and immune cell receptors are stable and can activate the immune system. MMGBSA of −146.27 net energy (kcal/mol) was calculated for the vaccine–TLR2 complex, while vaccine–TLR4 of −148.11 net energy (kcal/mol) was estimated. Furthermore, the C-ImmSim bioinformatics tool predicted that the vaccine construct can activate the immune system against *F. hepatica*, eradicate the infection caused by *F. hepatica*, and reduce financial losses that need to be spent while protecting against infections of *F. hepatica.* The computational immune simulation unveils that the vaccine model can activate the immune system against F*. hepatica*; hence, the experimental scientist can validate the finding accomplished through computational approaches.

## Introduction

*Fasciola hepatica* is one of the leading causes of fasciolosis in animals and humans (1). Along with *Fasciola gigantica*, it is a widely disseminated species of liver fluke. A major zoonotic trematode parasite instigates food-borne fasciolosis in livestock and humans. Adult *F. hepatica* fluke are flatworms, measuring 20–40 mm long and 8–13mm wide (2). Domestic ruminants of tropical and temperate regions are susceptible to the disease caused by *F. hepatica*. The worm/parasite can infect new hosts, such as wild animals, contributing to its worldwide transmission. Traditionally, fasciolosis is known as livestock disease, but it has become a significant emerging disease in humans (3).

The epidemiological surveys reported that human fasciolosis occurs in the region where the animal fascioliasis is endemic. The number of human cases reported was <3,000 before 1992, whereas *F. hepatica* infected approximately 17 million people worldwide in 2005. Furthermore, more than 91.1 million people were considered to have the risk of developing liver fluke infection. On the contrary, some countries, such as China, have rare cases of human infection, although veterinary *F. hepatica* infection is of considerable importance (4).

Fascioliasis due to *F. hepatica* poses a notable threat to the growth of the farming industry and public health in developing and developed countries. The continuous rise in morbidity and mortality cases led researchers to come up with solutions to combat life-threatening parasitic infections (5).

Climate change and environmental conditions play a critical role in the life cycle and transmittance of *F. hepatica*. Humidity, oxygen tension, and vegetative conditions also affect the liver fluke lifecycle. These factors provide favorable conditions for the development and reproduction of its larvae. The helminth is endemic in areas with a mean temperature above 10°C for 6 months, with reports of snail infections (6). The incidence of infection in humans is also severely aggregated by the dietary habits of the individuals who intake aquatic plants during animal husbandry (7).

The large leaf-shaped endoparasite has an intermediate host, *Lymnaea*, a freshwater snail, while the definitive hosts of *F. hepatica* are goats, cattle, sheep, and humans (8). Parasites reside in the bile duct of the mammalian liver, where their eggs leave the host through feces after entering the duodenum. The optimum conditions promote the growth of ciliated larvae (miracidium) inside the egg within 2–3 weeks. The larvae then escape from the egg and swim to reach the intermediate host, freshwater snail (*Lymnaea truncatula*) (9). Once it gets penetrated, it forms a sporocyst by losing ciliated covering. The germinal cells inside the sporocyst grow and divide to form the redia. The sporocyst then burst out with the growth of rediae to mature into the final larval form known as cercariae (10). The cercariae have a large tail, which helps it to leave the snail and swim in water to settle on aquatic plants within 2 h. The larva loses its tail afterwards to form metacercariae, which cause infection when animals and humans ingest them. The host’s intestinal fluid digests the metacercariae’s cyst wall to release the juvenile flukes. The flukes of *F. hepatica* become highly infective when they reach the liver within 4–6 days (11).

Consequently, they cause fascioliasis when they arrive in liver parenchymal cells in 5–6 weeks. The flukes lay eggs into the bile duct after getting sexually matured on week 7 after infection. The eggs leave the definitive host on week 8 after infection through the bile duct and in feces. Humans allow juvenile flukes to mature within 3–4 months (12).

The signs and symptoms of human fascioliasis are divided into two stages: the hepatic phase, which lasts 1–3 months, and the biliary phase. During the hepatic phase, patients experience fever, abdominal pain, cramps, eosinophilia, and abnormal liver function tests. The biliary phase of *F. hepatica* results in cholestasis with right upper quadrant pain in infected patients. The hepatic phase is diagnosed by computer tomography imaging (CT scan), while ultrasonographic methods detect the biliary phase of *F. hepatica*. Infected patients’ stool and blood samples are taken to confirm fascioliasis (13). Fascioliasis is also a zoonotic infection that can infect humans and is considered a significant source of morbidity and mortality rate (14).

Anemia, malnutrition, liver abscess, liver cirrhosis, and liver fibrosis are the complications seen in patients with acute and chronic infections. Triclabendazole is a drug that treats acute and chronic human fascioliasis (15). However, liver flukes of *F. hepatica* have developed resistance against Triclabendazole. Various antigens and biomarkers have been discovered to detect the resistance that has emerged from this drug (16). Re-purposing anti-helminthic drugs, such as nitroxinil, albendazole, and closantel, is in progress to effectively treat flukicide-resistant infections. Moreover, plant extracts and multiomic studies propagated the discovery of new targets and drugs to combat the rapidly spreading infection. All the treatments go a long way to surpass clinical trials, as they are seen to be successful only in laboratory practice. Lastly, preventive measures should be adopted to control the *F. hepatica* fluke (16).

Fluke control is impossible with the preventive measures and the above treatment options; therefore, effective vaccine development is crucial. Although various vaccination assays have been developed previously, only a few were tested in animals to evaluate their efficacy against fascioliasis. The candidate antigens, including the native and recombinant antigens secreted from *F. hepatica*, were checked to assess their effectiveness. The vaccines were based on cathepsins secreted from juvenile *F. hepatica* worms. The results obtained from clinical trials of the vaccines were not satisfactory (17). Hence, multi-epitopes vaccine is needed as a promising treatment for human and livestock fascioliasis. This study aims to utilize multi-informatics methods to design an *in silico*-based vaccine against *F. hepatica*.

## Research methodology

### Sequence retrieval of target protein selection

**Figure 1:**
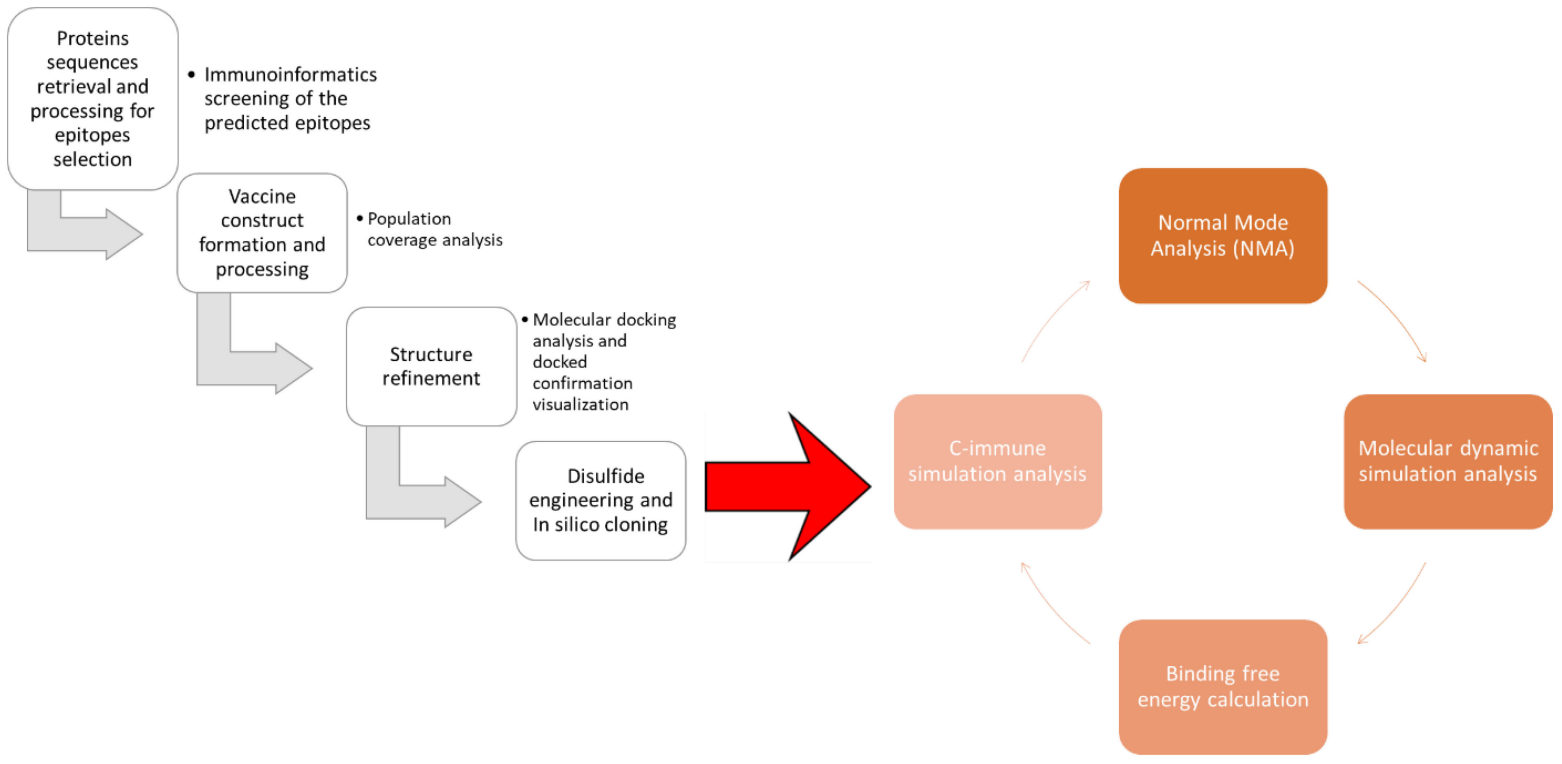
A diagrammatic depiction of the design process for a multi-epitope vaccine.

The protein sequence of the glutathione transferase and Cathepsin L-like proteinase was retrieved from UniProtKB by using ID A0A890CT21 and Q24940 (18). The target protein sequence was subjected to antigenicity analysis; antigenicity was by Vaxijen 2.0 server by selecting a target organism as a parasite with a threshold value of 0.4 (19). Furthermore, the physiochemical properties analysis and allergenicity were done through AllerTOP v. 2.0 and the expasy tools (20). **Figure 1** depicts the steps followed to design epitope based vaccine against Fasciola hepatica.

### Assessment and prediction of epitopes

The immune epitope database and tool (IEDB) sequence base searching tool for exposing and searching immune epitopes was utilized for the forecasting of epitopes in targeted protein sequences; in B-cell epitopes targeting, a piped linear epitope prediction 2.0 tool was used, and a set of full HLA alleles were selected (21). After B-cell epitope selection, T-cell epitopes were predicted from B-cell epitopes (22). The predicted epitopes were ranked based on the lowest percentile scoring and best overlapping. The predicted epitopes were subjected to allergenicity, toxicity, and water solubility analysis using Vaxijen (23), ToxinPred (24), and peptide calculator bioinformatics tools, respectively (25), and only non-toxic, non-allergen, and suitable water-soluble epitopes were selected.

### Designing and processing of vaccine construct

The chosen epitopes were utilized in developing the epitope vaccine construct; the epitopes were linked by “GPGPG” connectors (26). Furthermore, the model vaccine was combined with the cholera toxin B subunit adjuvant to enhance its potency (27). After making the linear sequence of the model vaccine, its physiochemical properties were analyzed using the protparam tool. Afterwards, physiochemical properties analysis and immunoinformatics screening were done using Vaxijen 2.0, peptide calculator, and Allertop 2.0 bioinformatics tools (28). After immunoinformatics screening, the sequence was used in 3D structure modeling using an online 3D scratch predictor bioinformatics tool (29). The model vaccine was subjected to loop refinement using the galaxy web refinement tool https://bio.tools/galaxyrefine. After the refinement of the vaccine structure, the structure was subjected to disulfide engineering by design 2.0, and the engineered structure was generated (30). The world population coverage analysis of the selected epitopes was predicted using the population analysis coverage analysis tool (27, 31, 32) in the immune epitopes database resources tool (33).

### Molecular base interaction of vaccine and immune cell receptor, molecular dynamic simulation analysis, and binding free energies estimation

The molecular docking analysis was used to evaluate the binding affinity and interaction pattern of the vaccine construct with receptor TLR2 and TLR4 using their specific PDB ID (TLR2-PDB: ID2Z7X and TLR4-PDB: 4FXI); the crystal structure of the target receptor was retrieved from Protein Data Bank PDB (34). After receptor retrieval, the vaccine construct and receptor were uploaded into the protein–protein molecular docking ClusPro 2.0 web tool (35). Furthermore, the interactive residues of the docking complexes were validated through the PDBsum tool (36). After molecular docking analysis, the docking complexes were subjected to molecular dynamic simulation analysis of 500-ns simulation time (37). In simulation investigation, root mean square fluctuation (RMSF) and root mean square deviation (RMSD) were calculated, using AMBER21 packages (38). Furthermore, using the iMODS server, the motion and stability of the docked conformation were evaluated (39). To further confirm the docking results and the docked complexes’ stability, the complexes’ binding free energy was assessed using MMGBSA and MMPBSA analyses (40).

## Results

### Protein sequences retrieval and processing for epitope selection

*Fasciola hepatica* glutathione transferase and Cathepsin L1 protein sequences were retrieved from UniProtKB using A0A890CT21 and Q24940 uniprotkb_accession numbers, respectively. The Vaxijen 2.0 webserver predicted both the proteins as probable antigenic with 0.4576 and 0.5697 antigenic values, respectively; next, the Allerton 2.0 bioinformatics tool predicted both the proteins as non-allergic. The ExPASy ProtParam tool predicted 218, 25.40237, 7.63, and −0.388 number of amino acids, molecular weight, theoretical pI, and GRAVY, respectively, for glutathione transferase, while for Cathepsin L1, the number of amino acids, molecular weight, theoretical pI, and GRAVY of 326, 36.89645, 6.71, and −0.503 were calculated, respectively. In the physiochemical properties analysis, we observed that both the selected proteins are physicochemically stable in the nature and subject for epitopes prediction and prioritization. In the epitopes selection and prioritization phase, the first B-cell epitopes were predicted, as mentioned in **Table 1**.

**Table 1:** Predicted B-cell epitopes.

Glutathione transferase	B-cell peptide
	EEYAERRYGQEEFGRWQQEKCTIDLD
	SPQLEEEKKKLLE
	KRIEDLPPIKKYMNSDRFIKWPLQAWFAGFGGGSA
**Cathepsin L1**	LWHQWKRMYNKEYNGADDQHRRNIWEKNVKHIQEHNLRHDLGL
	TDMTFEEFKAKYLTEMSRASDILSHGVPYEANNRAVPDKIDWRESGYVTEVKDQG
	LKQFGLETESSYPYTAVEGQCRYNKQLGVAK
	FMMYRSGIYQSQTCSPL

The predicted B-cell epitopes were utilized for MHC-I and MHC-II epitopes to make the vaccine construct to activate both humoral and cellular immunity against the target pathogens; the MHC-I and MHC-II predicted epitopes are shown in **Table 2**.

**Table 2:** T cells, MHC I, and MHC II predicted epitopes with target alleles.

MHC-I	Allele	Percentile score	MHC-II	Allele	Percentile score
AERRYGQEEF	HLA-A01:01	0.14	ERRYGQEEFGRWQQE	HLA-DRB3*01:01	23
GRWQQEKCTI	HLA-A*24:02	5.6	EFGRWQQEKCTIDLD	HLA-DRB3*01:01	32
LEEEKKKLL	HLA-B*40:01	0.2	PQLEEEKKKLL	HLA-DRB1*03:01	3.5
IEDLPPIKKY	HLA-B*44:03	0.02	IKKYMNSDR	HLA-DRB1*15:01	1.6
QEHNLRHDL	HLA-B*40:01	0.09	YNGADDQHR	HLA-DRB5*01:01	0.67
RNIWEKNVKH	HLA-A*03:01	0.9	LWHQWKRMYNKEYNG	HLA-DRB5*01:01	5.5
EYNGADDQHR	HLA-A*33:01	0.4	RRNIWEKNVKHIQEH	HLA-DRB3*02:02	6
WHQWKRMYNK	HLA-A*03:01	4.5	VKHIQEHNLRHDLGL	HLA-DRB4*01:01	23
RAVPDKIDW	HLA-B*58:01	0.01	LSHGVPYEA	HLA-DQA1*05:01/DQB1*03:01	0.48
RESGYVTEVK	HLA-A*11:01	1.9	VPYEANNRAVPDKID	HLA-DRB1*11:01	3.2
LSHGVPYEAN	HLA-B*58:01	13	WRESGYVTE	HLA-DPA1*01:03/DPB1*04:01	2.9
YLTEMSRASD	HLA-A*01:01	23	FKAKYLTEMSRASDI	HLA-DRB1*04:01	0.9
TDMTFEEFKA	HLA-A*02:01	13	TDMTFEEFKAKYLTE	HLA-DQA1*05:01/DQB1*03:01	0.68
ESSYPYTAV	HLA-A*68:02	0.02	YTAVEGQCR	HLA-DRB5*01:01	0.34
LKQFGLETES	HLA-A*02:06	21	YNKQLGVAK	HLA-DRB5*01:01	17
CRYNKQLGVA	HLA-A*30:01	23	SSYPYTAVEGQCRYN	HLA-DRB5*01:01	0.34
FMMYRSGIY	HLA-B*15:01	0.2	YRSGIYQSQ	HLA-DPA1*01:03/DPB1*02:01	15

### Immunoinformatics screening

In immunoinformatics screening, the antigenicity, allergenicity, water solubility, and toxicity of the predicted epitopes were analyzed, and only antigenic, non-allergic, non-toxic, and suitable water-soluble epitopes were shortlisted for vaccine designing; the shortlisted epitopes and antigenic values and other immunoinformatics parameters are mentioned in **Table 3**.

**Table 3:** Shortlisted immunoinformatics-filtered epitopes for vaccine designing.

B-cell-derived T-cell epitopes	Target allele	Percentile score	Antigenicity	Allergenicity	Water solubility	Toxicity	IFN-γ inducer
EFGRWQQEKCTIDLD	HLA-DRB3*01:01	32	0.6564	PROBABLE NON-ALLERGEN PROBABLE	Good water solubility.	Non-toxic	Positive
RRNIWEKNVKHIQEH	HLA-DRB3*02:02	6	0.5442				
FKAKYLTEMSRASDI	HLA-DRB1*04:01	0.9	1.1014				
TDMTFEEFKAKYLTE	HLA-DQA1*05:01/DQB1*03:01	0.68	1.1035				
YTAVEGQCR	HLA-DRB5*01:01	0.34	0.8005				

### Analysis of worldwide population coverage

The population coverage analysis predicted that the selected epitopes have shown coverage of 99.74% of the worldwide population, and 99.46%, 58.09%, 99.18%, 99.98%, 99.71%, 99.79%, and 99.88% of United States Asian, United States Austronesian, United States Black, United States Caucasoid, United States Hispanic, United States Mestizo, and United States Polynesian, respectively. The other countries and its worldwide population harbor are mentioned in **Supplementary Table S1**.

### Vaccine construct formation and processing

In the vaccine construction phase, the selected epitopes were linked with the help of a GPGPG linker and attached with an adjuvant through another linker, the EAAAK linker; the cholera toxin B subunit was used as an adjuvant. After vaccine construction, the physiochemical properties of the vaccine construct were analyzed through the ExPASy ProtParam tool. The server predicted 234 amino acids, 25,862.57 kDa molecular weight, 8.54 theoretical pI, 36.86 instability index, and −0.424 grand average of hydropathicity (GRAVY); the ExPASy ProtParam tool showed that the model vaccine is stable. Furthermore, the Vaxijen 2.0 bioinformatics tool evaluates the model vaccine as a probable antigen with 0.5000 (probable ANTIGEN) predicted value. Moreover, the Allertop 2.0 tool predicted that the sequence of the model vaccine is non-antigenic by nature. The peptide solubility calculator and ToxinPred 2.0 bioinformatics tools predicted that the sequence of the vaccine construct is good, water soluble, and non-toxic in nature. The physiochemical properties and immunoinformatics findings are mentioned in **Table 4**.

**Table 4:** Physiochemical properties and immunoinformatics analysis of model vaccine.

Multi-epitopes vaccine construct	Number of amino acids	Molecular weight	Theoretical pI	Instability index	GRAVY
234	25,862.57	8.54	36.86	−0.424
**Immunogenicity**	**Allergenicity**	**Hydrophilicity**	**Toxicity**	
Probable immunogenic	Non-allergic	Water soluble	Non-toxic	

**Figure 2:**
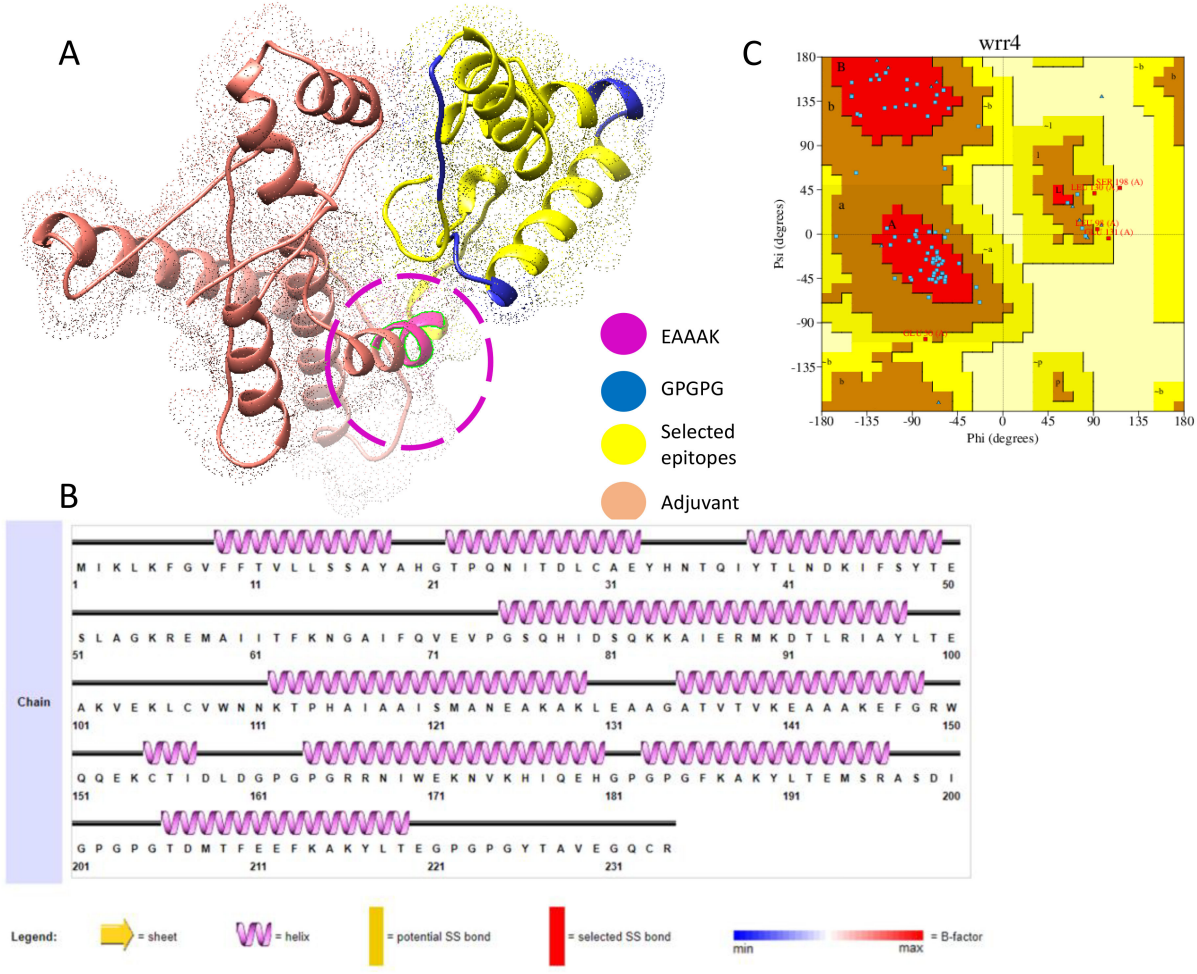
**(A)** 3D representation of multi epitopes vaccine construct. **(B)** Secondary structure. **(C)** Ramachandran plot.

**Figure 2A** shows the 3D structure of the engineered vaccine construct; the salmon color represents the adjuvant. Furthermore, the Ramachandran plot and secondary structure are mentioned in **Figures 2B, C**, respectively.

### Structure refinement

**Figure 3:**
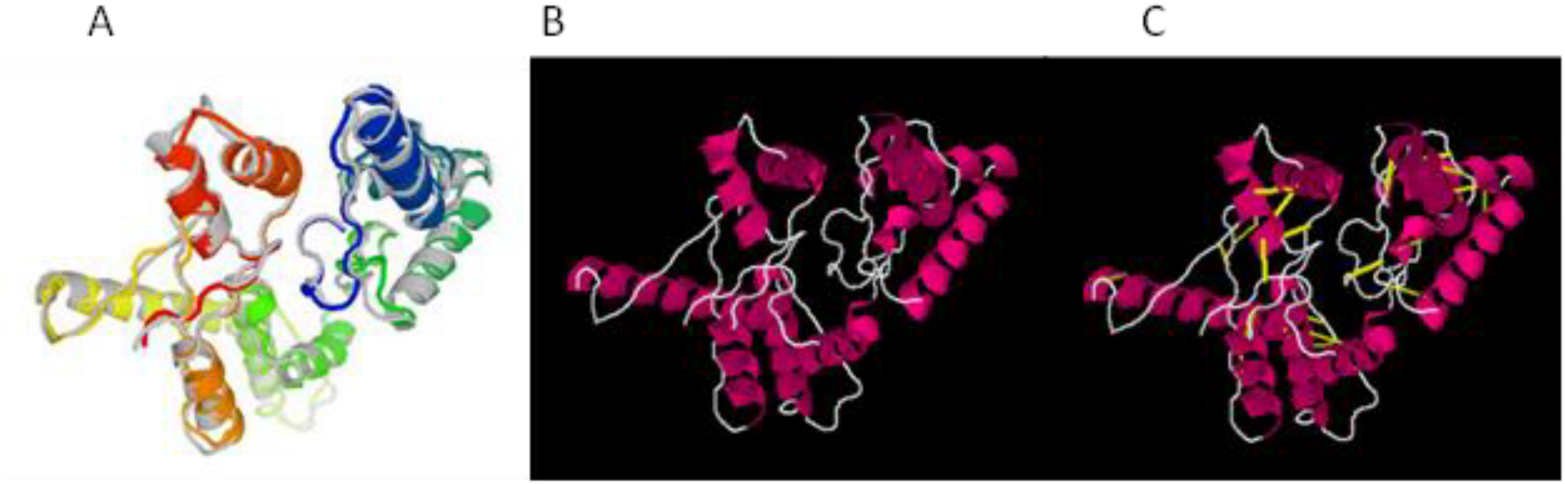
**(A)** Refine structure. **(B)** Original structure. **(C)** Mutated structure.

The structure of the vaccine construct was refined to remove the unwanted loops in the protein structure, and overall, the steric clashes were removed. The refined structure is presented in **Figure 3A**. The galaxy refinement tool generated the top 10 refine structure based on several parameters, and the calculated values are mentioned in **Supplementary Table S2**. The model refine structure was considered deemed fit for further processing. For further improvement, disulfide engineering was performed on the structure of the vaccine construct, in which disulfide bonds were incorporated in the refine structure of the vaccine construct. The vaccine construct was further subjected to disulfide engineering, and a total of 25 pairs of amino acid residues Leu4-Thr11, Gly7-Val229, Val8-Thr11, Phe9-His34, Val12-Thr27, Ser15-Pro23, Ile384-Leu41, Lys44-Lys55, Met58-Asn65, Ala59-Thr62, Pro74-Gln77, Ile95-Ser121, Leu98-Lys102, Val103-Ile120, Ala123-Glu141, Lys154-Ile157, Asp160-Arg166, Ile177-Phe186, Tyr190-Pro204, Leu191-Gly203, Asp199-Pro202, Pro202-Glu212, Gly205-Met108, Tyr217-Gly223, and Tyr226-Val229 were chosen to be replaced with cysteine amino acid and the refined structure generated; the original and mutated structures are presented in **Figures 3B, C**.

### Molecular docking analysis and docked confirmation visualization

**Figure 4:**
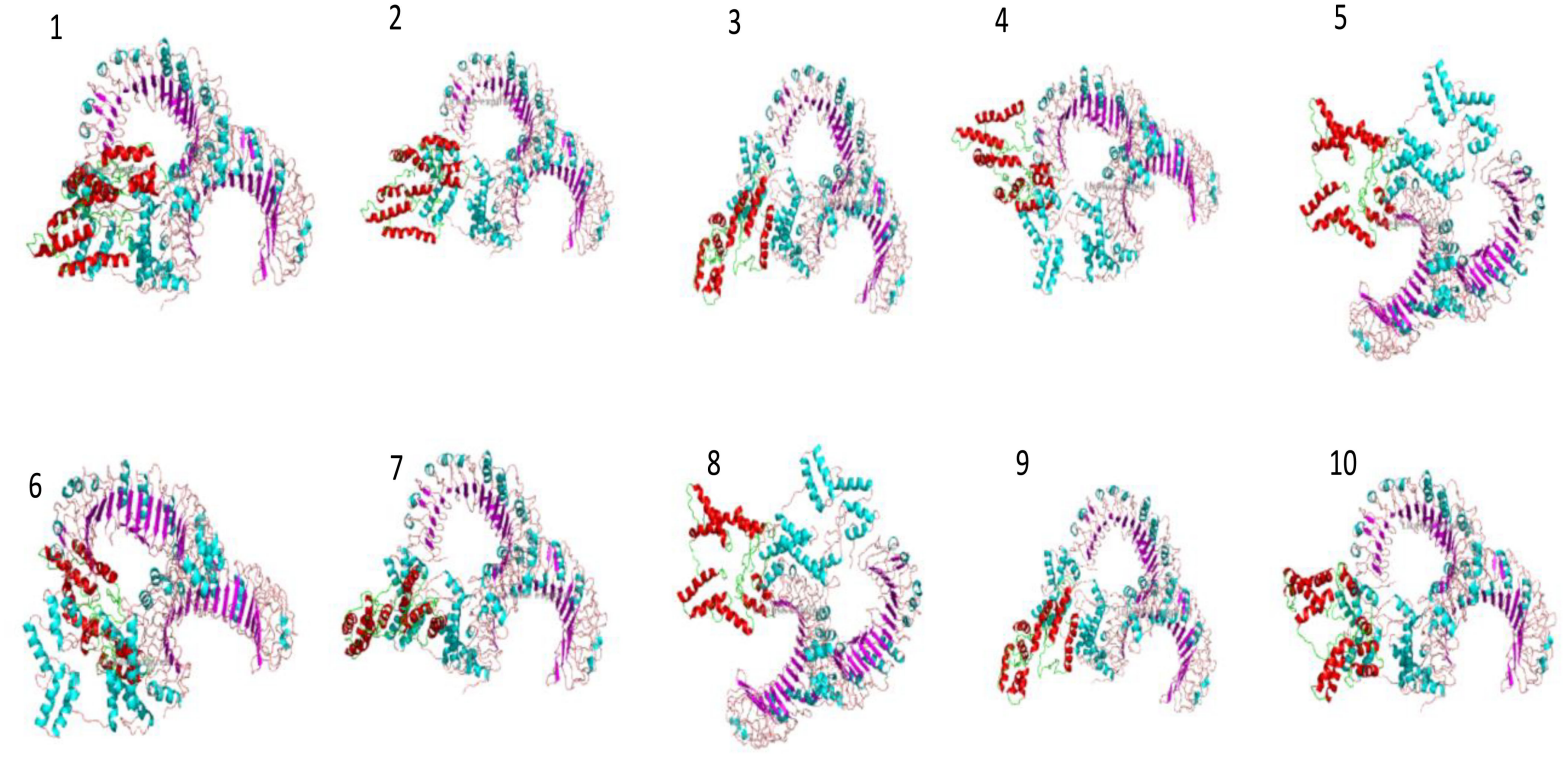
Intermolecular docked confirmation of vaccine and TLR2.

**Figure 5:**
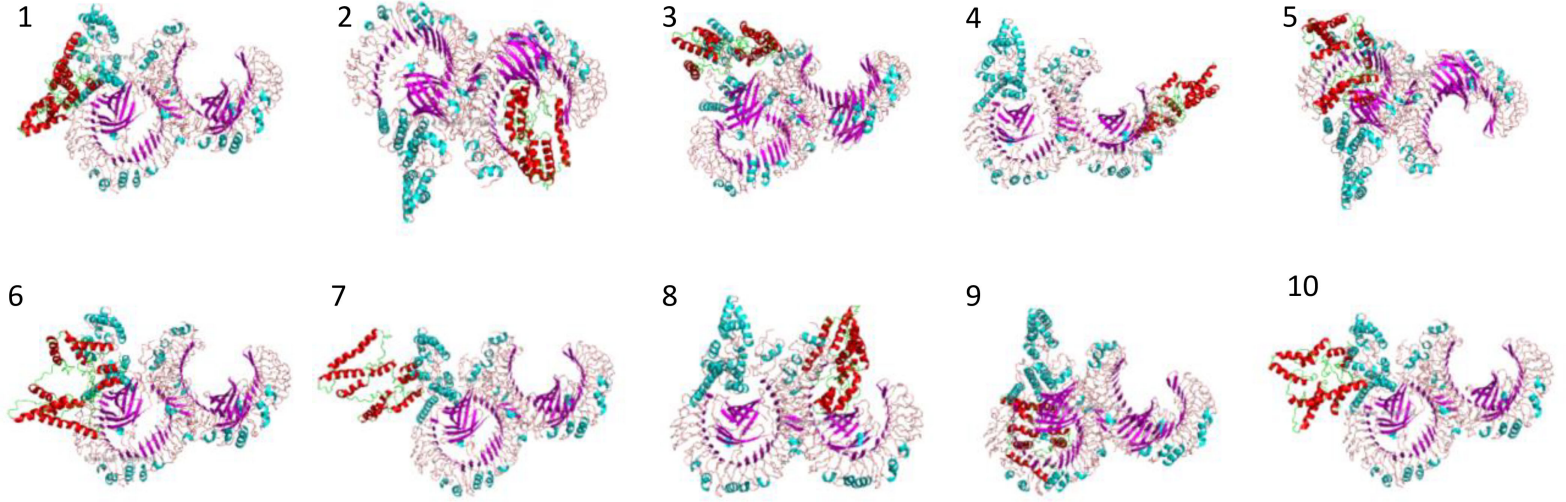
Intermolecular docking conformation of the vaccine and TLR4.

In molecular docking analysis, we observed the vaccine’s interaction with TLR2 and TLR4. The docking served different binding energy scores between the vaccine and receptor, and the energy was predicted based on the blinding docking approach. The docking scores of the vaccine and TLR2 and TLR4 are presented in **Supplementary Tables S3, S4**, respectively. The intermolecular docking visualizations are shown in **Figures 4**, **5**.

### Intermolecular visualization of docked molecules

**Figure 6:**
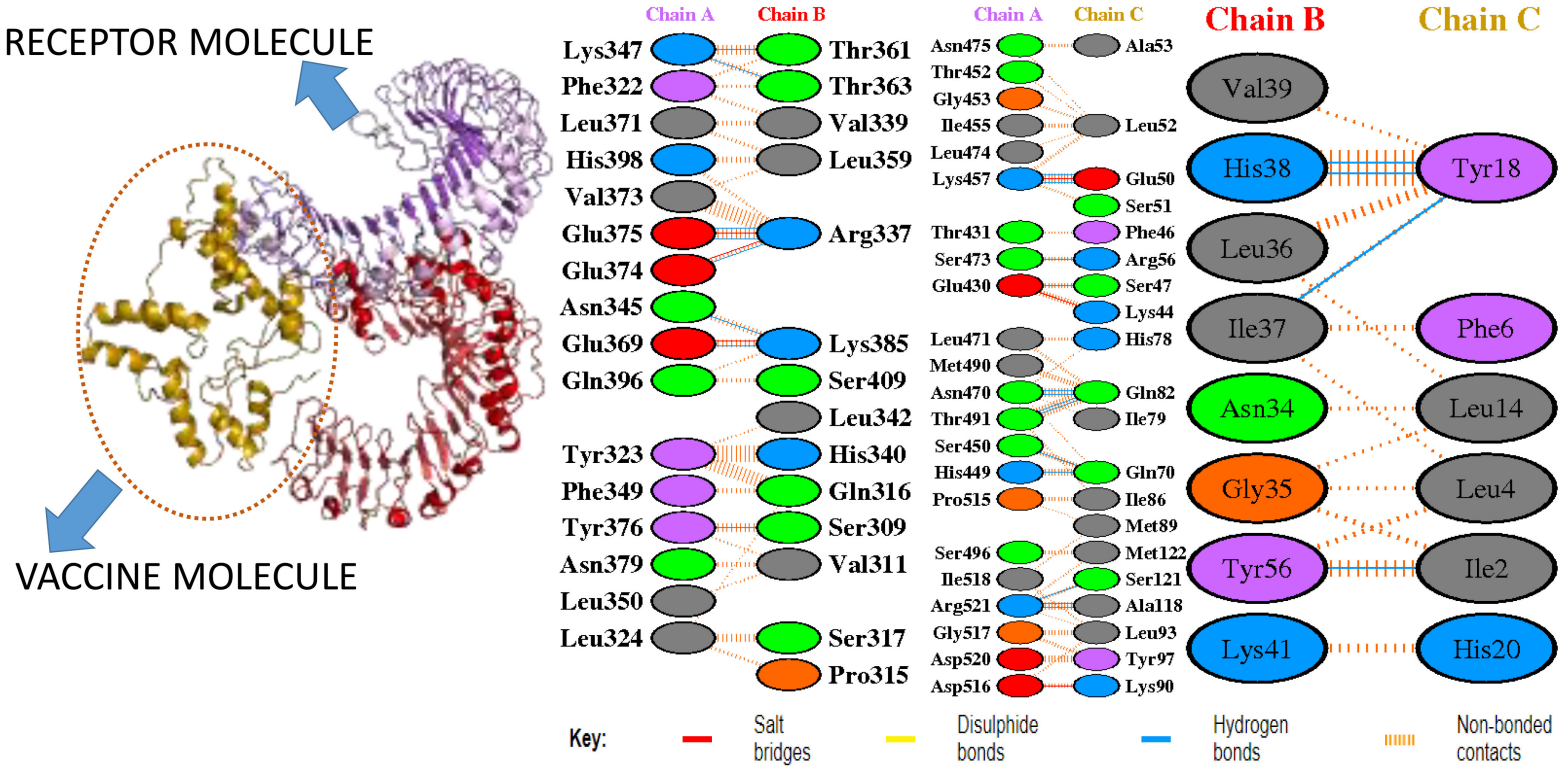
Bonding interaction of vaccine and TLR2.

**Figure 7:**
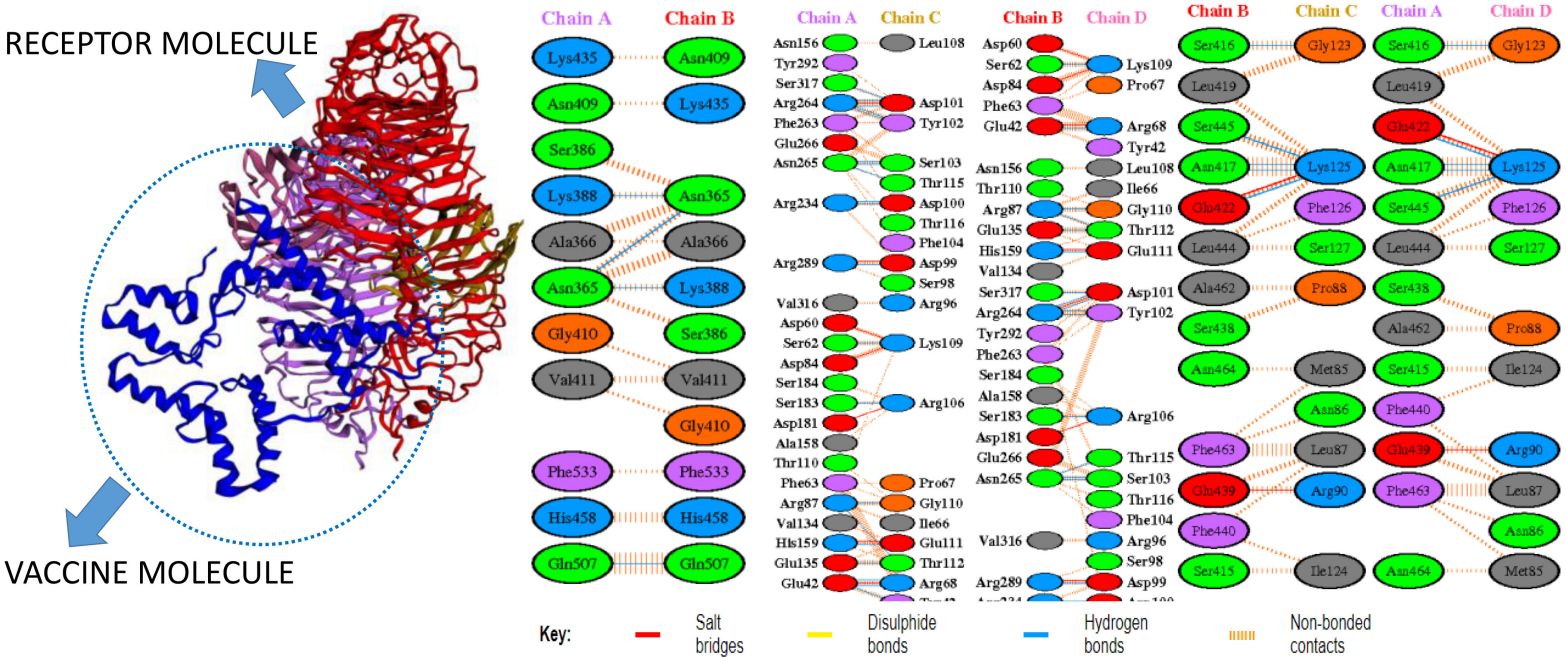
Bonding interaction of vaccine and TLR4.

The intermolecular interaction of top 1 docked complexes, based on different amino acid residue levels, is visualized by pdbsum generated in the amino acid residues. Other types of bonding interactions have been observed in vaccine and TLR2 and TLR4 docked complexes, as presented in **Figures 6**, **7**.

### iMODS analysis

**Figure 8:**
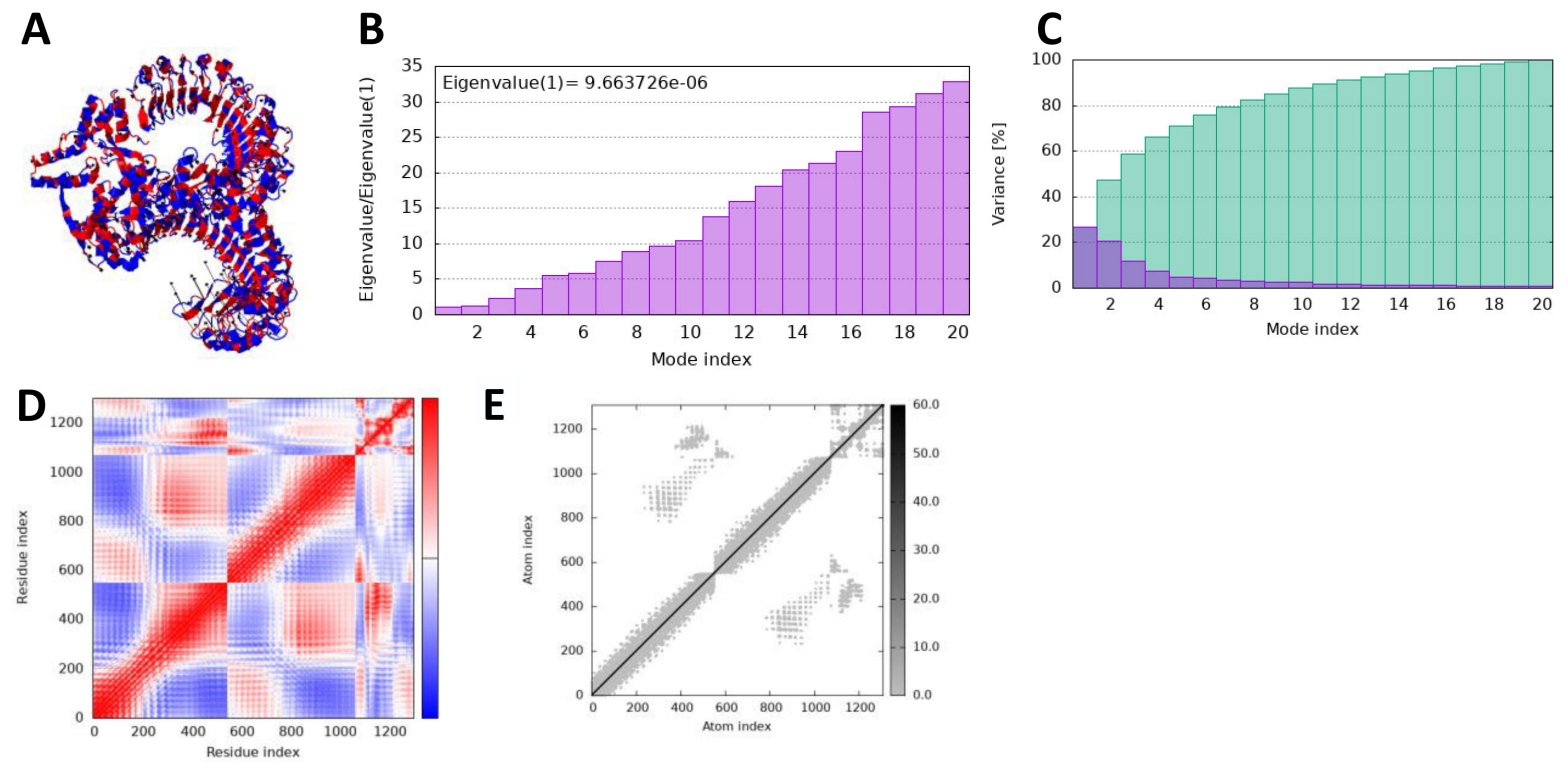
iMODS simulation of vaccine_TLR4. **(A)** Docked structure. **(B)** Eigenvalue. **(C)** Variance. **(D)** Covaiance. **(E)** Elastic network model.

**Figure 9:**
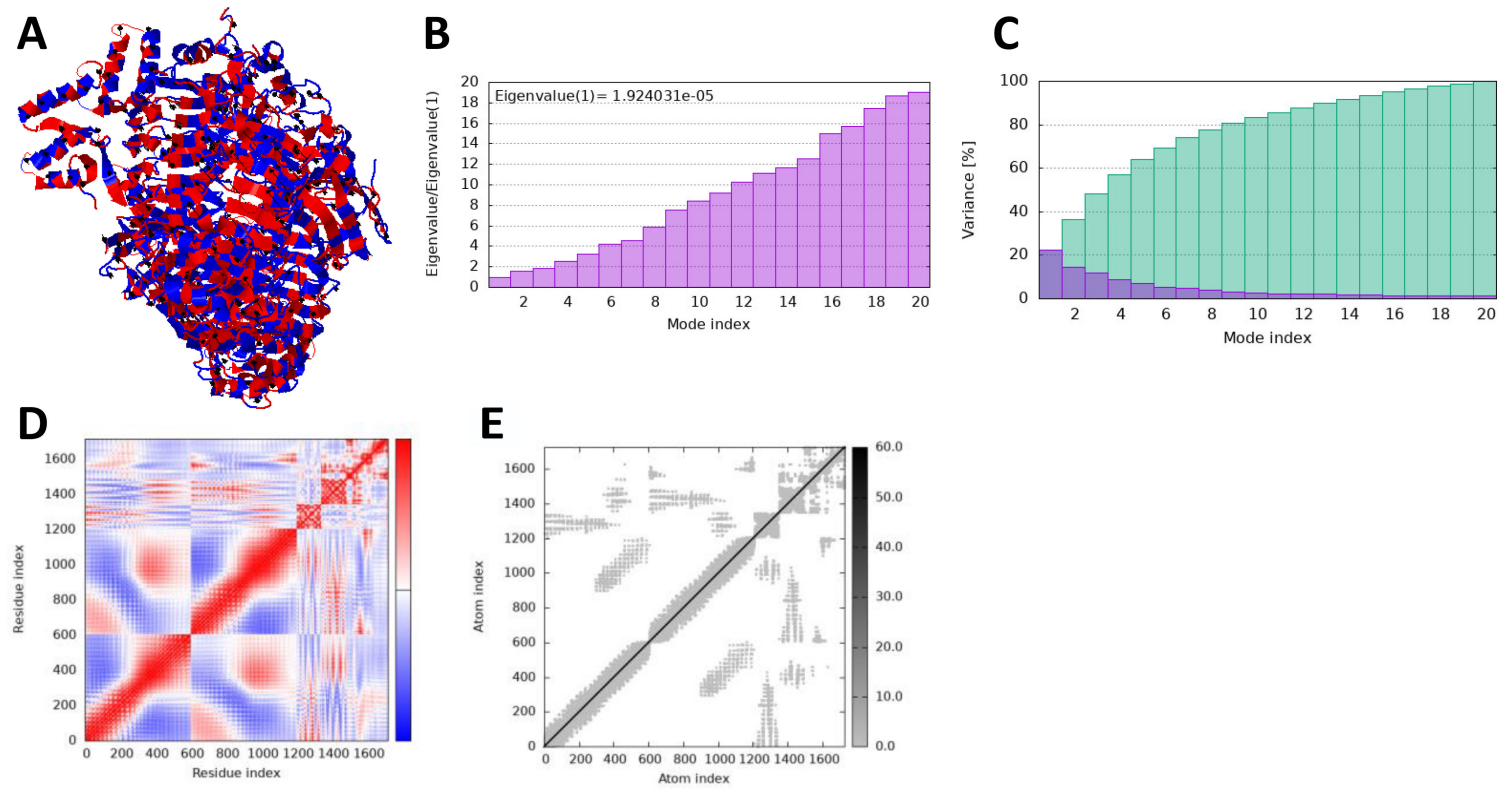
iMODS simulation of vaccine_TLR4. **(A)** Docked structure. **(B)** Eigenvalue. **(C)** Variance. **(D)** Covaiance. **(E)** Elastic network model.

In iMODS simulation, the vibration motion of the docked complexes is increasingly used to study the dynamics of the docked complexes; we predicted eigenvalues of 9.663726 and 1.924031 in the case of vaccine and TLR2 and TLR4, respectively, which correlate that docked complexes have been showing stability in the dynamic environment. Furthermore, the residues and atomic indices also showed that the vaccine and receptors presented stable docked molecules, as the normal modes analysis plots are mentioned in **Figures 8**, **9**.

### MD simulation analysis

**Figure 10:**
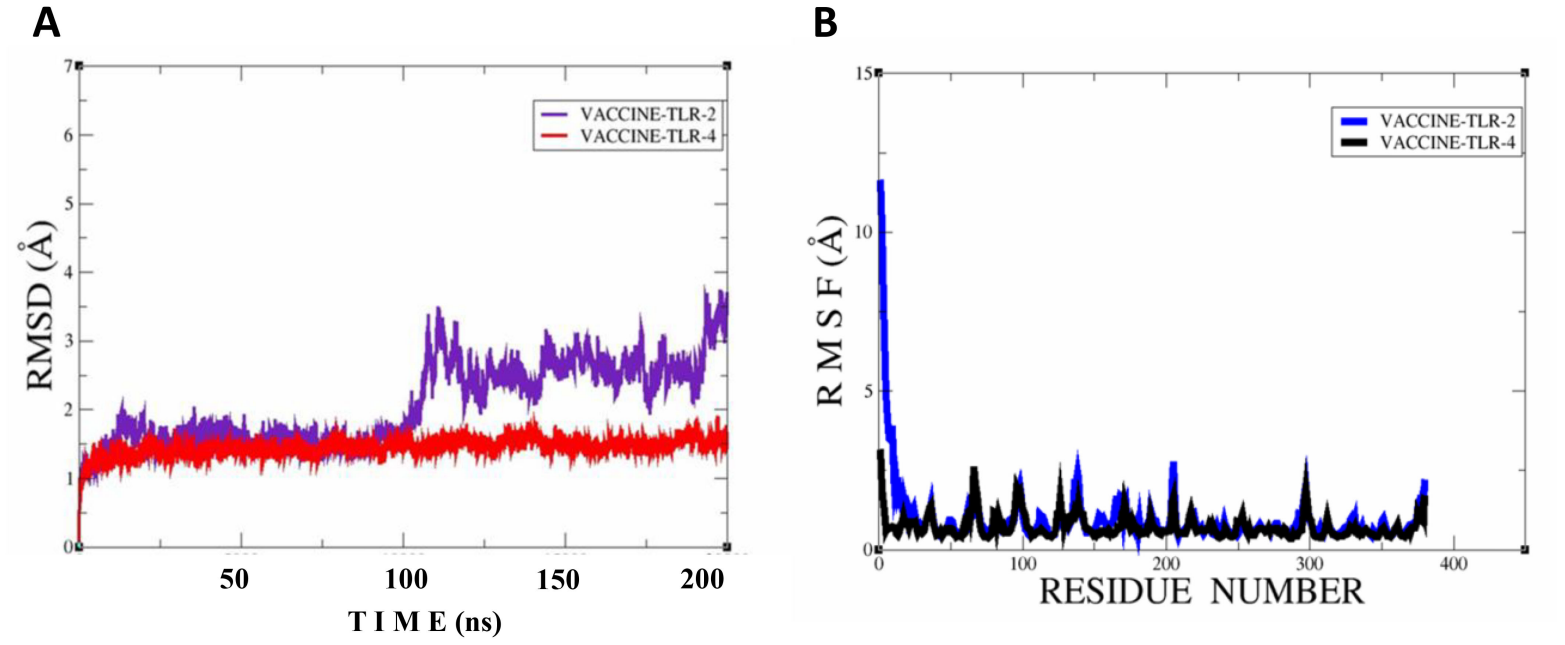
Simulation plots of vaccine–TLR2 and vaccine–TLR4. **(A)** RMSD plot for both docked complexes; vaccine-TLR2 and vaccine-TLR4. **(B)** RMSF plot for both docked complexes; vaccine-TLR2 and vaccine-TLR4.

In the molecular dynamic simulation, the plot represents that the vaccine and the immune cell receptor stability remains in the dynamic environment; root mean square deviation analysis was done to observe time-dependent deviation in docked complexes, while root mean square deviation was done to achieve residue level fluctuation in the docked molecules. In the RMSD, we noticed that the vaccine–TLR4 is more stable followed by vaccine–TLR2 in 200 simulation times. Moreover, the RMSF plot also represents that the immunization and TLR4 are more stable, followed by vaccine and TLR4, as shown in **Figures 10A, B**.

### Estimation of binding energy

The calculation of binding free energy predicted that the vaccine and receptor binding affinity is stable, and the MMGB/PBSA analysis predicted the negative binding energy, unveiling that the net binding energy is a negative value, hence representing that the complexes are stable. Negative biding energies of −148.11 and −146.27 were predicted via MMGBSA for vaccine–TLR4 and vaccine–TLR2 receptors, the MM-PBSA predicted −145.98 and −164.78, which was calculated by MMPBSA vaccine–TLR4 and vaccine–TLR2, respectively, as mentioned in **Table 5**.

**Table 5:** The binding energy of the vaccine and immune receptors.

Energy Parameter	Vaccine–TLR4	Vaccine–TLR2
MM-GBSA		
Van der Waals Energy (kcal/mol)	−140.21	−149.01
Columbic Energy (kcal/mol)	−33.14	−35.67
Total Gas Phase Energy (kcal/mol)	−161.35	−180.68
Total Solvation Energy (kcal/mol)	25.24	23.41
Net Energy (kcal/mol)	−148.11	−146.27
MM-PBSA		
Van der Waals Energy (kcal/mol)	−130.21	−145.01
Columbic Energy (kcal/mol)	−32.14	−33.67
Total Gas Phase Energy (kcal/mol)	−173.35	−170.68
Total Solvation Energy (kcal/mol)	20.37	20.90
Net Energy (kcal/mol)	−145.98	−164.78

### Computational immune simulation

**Figure 11:**
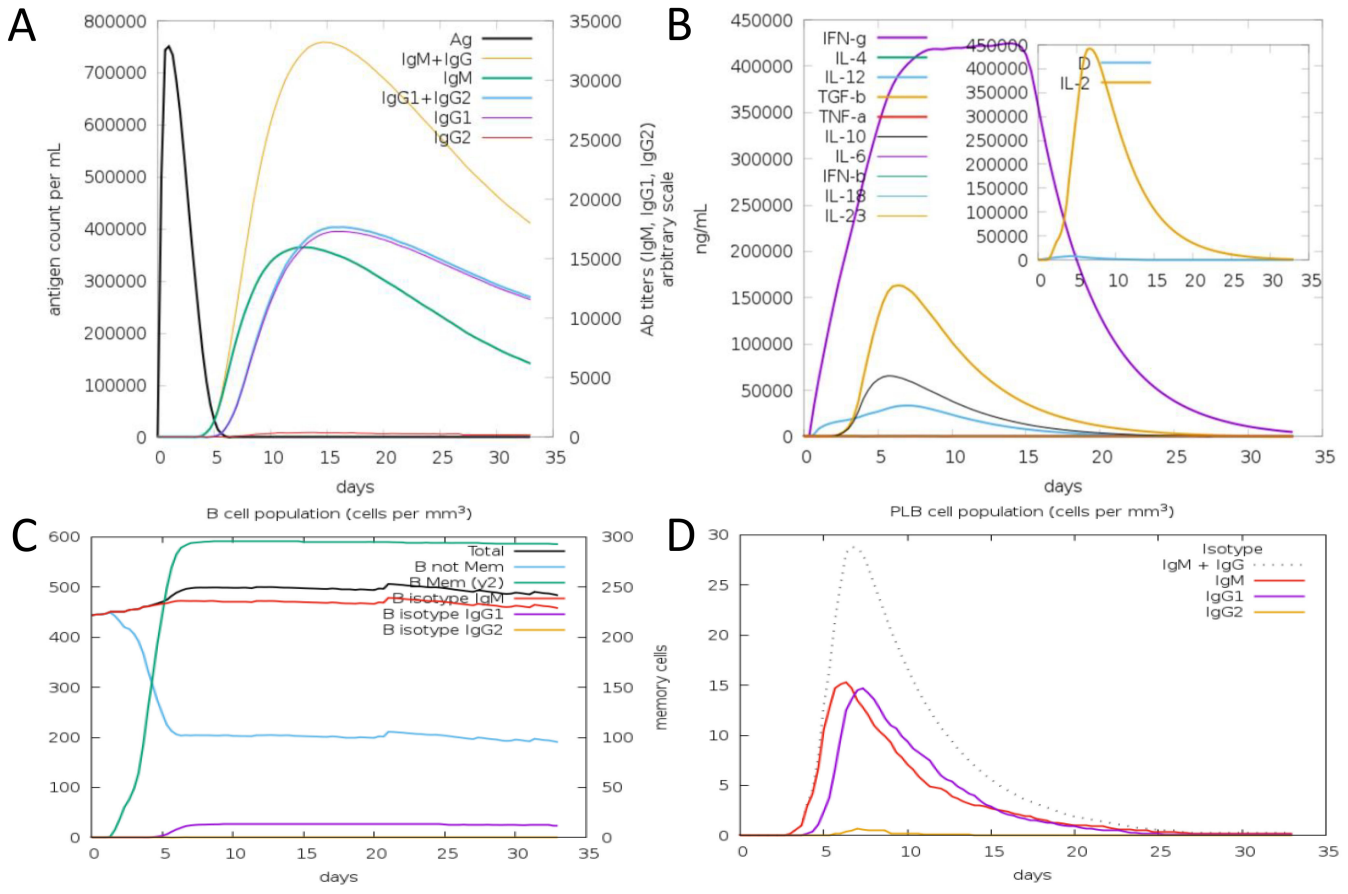
Antibodies and cytokines plots toward a model vaccine. **(A)** Antibody production level with number of days after vaccine. **(B)** Concentration of Cytokines and Interleukins against vaccine. **(C)** B cell population. **(D)** PLB cell population.

In computational immune simulation, we observed that the model vaccine against *F. hepatica* can activate the immune response in the host and efficiently tackle the pathogenesis. We analyzed different types of B and T cells’ immune response toward the *F. hepatica* vaccine, as presented in **Figures 11A-D**.

## Discussion

Vaccination is the primary method for preventing infections caused by pathogens (41). However, traditional vaccine development is expensive and labor intensive and carries a significant risk of failure (42). By contrast, immunoinformatics-driven vaccine design can streamline this process by pinpointing potential epitopes from proteins for use in creating vaccine candidates (43). Multi-epitope vaccines are crafted to enhance the collective impact of cellular, humoral, and innate immune responses, giving them an advantage over monovalent candidates or formulations (44). Recently, many efforts have been made to utilize immunoinformatics approaches for developing epitope vaccines against parasites (45).

In this research, a multi-epitope-based vaccine has been projected from two selected proteins, glutathione transferase and Cathepsin L-like proteinase, that play a significant role in the virulence and pathogenesis of parasites; the proteins were selected based on homology search, as they do not show homology with human proteins, which proposed a tremendously lesser possibility of causing autoimmune reactions in the host body (46). Unusually, the selected protein sequences were physicochemically stable, non-allergic, had good water solubility, non-toxic, and had 0 transmembrane helices (TM helices), indicating that the selected proteins are a promising vaccine candidate. Both types of host immunity, namely, the T-lymphocyte effector response and long-lasting B-cell memory, are crucial for protection against the parasite (47). Seven B-cell epitopes were predicted from both selected proteins to generate a humoral immune response against *F. hepatica*; from B cells, further T-cell epitopes were predicted to accomplish T-cell response as well (48).

Macrophage activation is essential for the elimination of microorganisms, and interferon-gamma is the main macrophage-activating factor. In the immunoinformatics analysis, we observed that the selected epitopes were found to be an IFN-γ activator (49). In addition, the B- and T-cell epitopes were also added in the vaccine construct for the activation of cellular- and humoral-mediated immune system *F. hepatica*. At the same time, none of the selected epitopes in the designed vaccine showed any similarity to the human proteome (50). This indicates its ability to trigger immune solid responses while avoiding potential harmful allergic reactions.

Keeping the synthetic protein small is essential. Minimizing the size of the artificial protein is vital to lower production costs, simplify the purification process from inclusion bodies, and avoid host organism toxicity. Therefore, only 10 epitopes were selected for the vaccine design, excluding overlapping ones, to optimize the total number of epitopes, without increasing the overall length (51).

Moreover, the docking analysis predicted that the immune system could recognize the model vaccine; hence, it can provoke the immune system and easily create a cellular and antibody-dependent immune system (52). In the dynamic environment, the stability of the docked complex is essential to activate and create long-lasting immunity against *F. hepatica*. The molecular dynamic simulation finding unveils the binding stability of the immune system and vaccine stability. Moreover, the *in silico* results estimated that the vaccine model could boost the immune system in the form of the cellular and humoral immune response (28, 29, 53, 54). The MMGBSA and MMPBSA analysis further validated that the vaccine and immune system have stable interaction. Overall, the findings suggest that the vaccine model can activate the immune system and can reduce the pathogenicity of the *F. hepatica*.

## Conclusion

Fasciolosis is not just a zoonotic infection and health issue but also affects social and economic values. Therefore, constructing a powerful and effective vaccine could be crucial in effectively combating this disease. This study mainly focused on epitope-based vaccine constructs against *F. hepatica* by precisely predicting possible vaccine targets from glutathione transferase and Cathepsin L-like proteinase. The developed vaccine construct exhibits acceptable properties regarding antigenicity, allergenicity, physicochemical characteristics, and structural integrity. Moreover, the interaction of the designed vaccine construct with the immune system indicates that the vaccine construct can activate the immune system and induce proper B- and T-cell response toward the *F. hepatica*; however, the findings further need to validate its potency against *F. hepatica*. It can be concluded that despite some structural changes observed after MD simulation, the vaccine construct remains stable *in vivo* within the biological system.

## Data Availability

The original contributions presented in the study are included in the article/**Supplementary Material**. Further inquiries can be directed to the corresponding author.
