# Supplementary material for: Exploring glutathione transferase and Cathepsin L-like proteinase for designing of epitopes-based vaccine against Fasciola hepatica by immunoinformatics and biophysics studies
Source: Front Immunol. 2024 Sep 26;15:1478107. doi: 10.3389/fimmu.2024.1478107 (PMC11464328; doi:10.3389/fimmu.2024.1478107)
Supplement: Supplementary file 1 [file Table1.docx]

**Exploring glutathione transferase and Cathepsin L-like proteinase for designing of epitopes based vaccine against *Fasciola hepatica* by immunoinformatics and biophysics studies**

Hassan H. Alhassan^1,*^, Muhammad Ikram Ullah^1^, Abdurahman A. Niazy^2^, Sami I. Alzarea^3^, Omar Awad Alsaidan^4^, Abdulaziz Ibrahim Alzarea^5^, Aseel Awad Alsaidan^6^, Abulaziz A. Alhassan^7^, Muharib Alruwaili^1^ ,Yasir S. Alruwaili^1^

^1^ Department of Clinical Laboratory Sciences, College of Applied Medical Sciences, Jouf University, Sakaka, Al-Jouf, 72388, Saudi Arabia; [h.alhasan@ju.edu.sa](mailto:h.alhasan@ju.edu.sa); [mikramullah@ju.edu.sa](mailto:mikramullah@ju.edu.sa); [ysalruwaili@ju.edu.sa](mailto:ysalruwaili@ju.edu.sa); [mfalrwaili@ju.edu.sa](mailto:mfalrwaili@ju.edu.sa)

^2^ Department of Oral Medicine and Diagnostic Sciences, College of Dentistry, King Saud University, Riyadh, 11451, Saudi Arabia; [aaniazy@ksu.edu.sa](mailto:aaniazy@ksu.edu.sa)

^3^ Department of Pharmacology, College of Pharmacy, Jouf University, Sakaka, Al-Jouf, 72388, Saudi Arabia; [samisz@ju.edu.sa](mailto:samisz@ju.edu.sa)

^4^ Department of Pharmaceutics, College of Pharmacy, Jouf University, Sakaka, Al-Jouf, 72388, Saudi Arabia; [osaidan@ju.edu.sa](mailto:osaidan@ju.edu.sa)

^5^ Clinical Pharmacy Department, College of Pharmacy, Jouf University, Sakaka, Al-Jouf, 72388, Saudi Arabia; [aizarea@ju.edu.sa](mailto:aizarea@ju.edu.sa)

^6^ Department of Family and Community Medicine, College of Medicine, Jouf University, Sakaka, Al-Jouf, 72388, Saudi Arabia, Saudi Arabia; aaalsaidan@ju.edu.sa

^7^ Department of Pediatric, Domat Aljandal General Hospital, Ministry of Health, Domat Aljandal, Al-Jouf, 72388, Saudi Arabia; [aaalhassan@moh.gov.sa](mailto:aaalhassan@moh.gov.sa)

***Corresponding author:** [h.alhasan@ju.edu.sa](mailto:h.alhasan@ju.edu.sa)

**Table S-1. Population coverage by selected epitopes**

| Worlds (countries ) | MHC-II | MHC-II | class combined |
| --- | --- | --- | --- |
| [Algeria](http://tools.iedb.org/population/result/#Algeria) | 0.00% | 77.15% | 77.15% |
| [Algeria Arab](http://tools.iedb.org/population/result/#Algeria%20Arab) | 0.00% | 77.15% | 77.15% |
| [American Samoa](http://tools.iedb.org/population/result/#American%20Samoa) | 98.75% | 0.00% | 98.75% |
| [American Samoa Polynesian](http://tools.iedb.org/population/result/#American%20Samoa%20Polynesian) | 98.75% | 0.00% | 98.75% |
| [Argentina](http://tools.iedb.org/population/result/#Argentina) | 97.50% | 62.67% | 99.07% |
| [Argentina Amerindian](http://tools.iedb.org/population/result/#Argentina%20Amerindian) | 97.50% | 45.78% | 98.64% |
| [Argentina Caucasoid](http://tools.iedb.org/population/result/#Argentina%20Caucasoid) | 0.00% | 80.65% | 80.65% |
| [Australia](http://tools.iedb.org/population/result/#Australia) | 94.51% | 33.15% | 96.33% |
| [Australia Australian Aborigines](http://tools.iedb.org/population/result/#Australia%20Australian%20Aborigines) | 87.31% | 33.15% | 91.52% |
| [Australia Caucasoid](http://tools.iedb.org/population/result/#Australia%20Caucasoid) | 99.94% | 0.00% | 99.94% |
| [Austria](http://tools.iedb.org/population/result/#Austria) | 99.91% | 93.34% | 99.99% |
| [Austria Caucasoid](http://tools.iedb.org/population/result/#Austria%20Caucasoid) | 99.91% | 93.34% | 99.99% |
| [Belarus](http://tools.iedb.org/population/result/#Belarus) | 0.00% | 43.81% | 43.81% |
| [Belarus Caucasoid](http://tools.iedb.org/population/result/#Belarus%20Caucasoid) | 0.00% | 43.81% | 43.81% |
| [Belgium](http://tools.iedb.org/population/result/#Belgium) | 99.39% | 79.39% | 99.87% |
| [Belgium Caucasoid](http://tools.iedb.org/population/result/#Belgium%20Caucasoid) | 99.39% | 79.39% | 99.87% |
| [Bolivia](http://tools.iedb.org/population/result/#Bolivia) | 0.00% | 77.82% | 77.82% |
| [Bolivia Amerindian](http://tools.iedb.org/population/result/#Bolivia%20Amerindian) | 0.00% | 77.82% | 77.82% |
| [Borneo](http://tools.iedb.org/population/result/#Borneo) | 0.00% | 49.02% | 49.02% |
| [Borneo Austronesian](http://tools.iedb.org/population/result/#Borneo%20Austronesian) | 0.00% | 49.02% | 49.02% |
| [Brazil](http://tools.iedb.org/population/result/#Brazil) | 96.10% | 63.80% | 98.59% |
| [Brazil Amerindian](http://tools.iedb.org/population/result/#Brazil%20Amerindian) | 93.24% | 48.60% | 96.52% |
| [Brazil Caucasoid](http://tools.iedb.org/population/result/#Brazil%20Caucasoid) | 99.32% | 84.39% | 99.89% |
| [Brazil Mixed](http://tools.iedb.org/population/result/#Brazil%20Mixed) | 97.02% | 77.50% | 99.33% |
| [Brazil Mulatto](http://tools.iedb.org/population/result/#Brazil%20Mulatto) | 0.00% | 74.09% | 74.09% |
| [Bulgaria](http://tools.iedb.org/population/result/#Bulgaria) | 99.42% | 57.23% | 99.75% |
| [Bulgaria Caucasoid](http://tools.iedb.org/population/result/#Bulgaria%20Caucasoid) | 99.52% | 57.23% | 99.79% |
| [Bulgaria Other](http://tools.iedb.org/population/result/#Bulgaria%20Other) | 99.58% | 0.00% | 99.58% |
| [Burkina Faso](http://tools.iedb.org/population/result/#Burkina%20Faso) | 67.18% | 0.00% | 67.18% |
| [Burkina Faso Black](http://tools.iedb.org/population/result/#Burkina%20Faso%20Black) | 67.18% | 0.00% | 67.18% |
| [Cameroon](http://tools.iedb.org/population/result/#Cameroon) | 89.72% | 49.87% | 94.85% |
| [Cameroon Black](http://tools.iedb.org/population/result/#Cameroon%20Black) | 89.72% | 49.87% | 94.85% |
| [Canada](http://tools.iedb.org/population/result/#Canada) | 0.00% | 38.41% | 38.41% |
| [Canada Amerindian](http://tools.iedb.org/population/result/#Canada%20Amerindian) | 0.00% | 38.41% | 38.41% |
| [Cape Verde](http://tools.iedb.org/population/result/#Cape%20Verde) | 99.18% | 80.38% | 99.84% |
| [Cape Verde Black](http://tools.iedb.org/population/result/#Cape%20Verde%20Black) | 99.18% | 80.38% | 99.84% |
| [Central Africa](http://tools.iedb.org/population/result/#Central%20Africa) | 86.04% | 62.71% | 94.79% |
| [Central African Republic](http://tools.iedb.org/population/result/#Central%20African%20Republic) | 28.02% | 82.69% | 87.54% |
| [Central African Republic Black](http://tools.iedb.org/population/result/#Central%20African%20Republic%20Black) | 28.02% | 82.69% | 87.54% |
| [Central America](http://tools.iedb.org/population/result/#Central%20America) | 7.76% | 49.91% | 53.80% |
| [Chile](http://tools.iedb.org/population/result/#Chile) | 95.94% | 67.08% | 98.66% |
| [Chile Amerindian](http://tools.iedb.org/population/result/#Chile%20Amerindian) | 99.63% | 72.65% | 99.90% |
| [Chile Mixed](http://tools.iedb.org/population/result/#Chile%20Mixed) | 90.09% | 52.65% | 95.31% |
| [China](http://tools.iedb.org/population/result/#China) | 94.57% | 59.99% | 97.83% |
| [China Oriental](http://tools.iedb.org/population/result/#China%20Oriental) | 94.57% | 59.99% | 97.83% |
| [Colombia](http://tools.iedb.org/population/result/#Colombia) | 8.36% | 54.02% | 57.86% |
| [Colombia Amerindian](http://tools.iedb.org/population/result/#Colombia%20Amerindian) | 0.00% | 47.40% | 47.40% |
| [Colombia Black](http://tools.iedb.org/population/result/#Colombia%20Black) | 3.65% | 65.25% | 66.51% |
| [Colombia Mestizo](http://tools.iedb.org/population/result/#Colombia%20Mestizo) | 14.07% | 56.31% | 62.45% |
| [Congo](http://tools.iedb.org/population/result/#Congo) | 0.00% | 68.66% | 68.66% |
| [Congo Black](http://tools.iedb.org/population/result/#Congo%20Black) | 0.00% | 68.66% | 68.66% |
| [Cook Islands](http://tools.iedb.org/population/result/#Cook%20Islands) | 0.00% | 78.59% | 78.59% |
| [Cook Islands Polynesian](http://tools.iedb.org/population/result/#Cook%20Islands%20Polynesian) | 0.00% | 78.59% | 78.59% |
| [Costa Rica](http://tools.iedb.org/population/result/#Costa%20Rica) | 0.00% | 24.31% | 24.31% |
| [Costa Rica Mestizo](http://tools.iedb.org/population/result/#Costa%20Rica%20Mestizo) | 0.00% | 24.31% | 24.31% |
| [Croatia](http://tools.iedb.org/population/result/#Croatia) | 99.79% | 66.71% | 99.93% |
| [Croatia Caucasoid](http://tools.iedb.org/population/result/#Croatia%20Caucasoid) | 99.79% | 66.71% | 99.93% |
| [Cuba](http://tools.iedb.org/population/result/#Cuba) | 98.92% | 85.48% | 99.84% |
| [Cuba Caucasoid](http://tools.iedb.org/population/result/#Cuba%20Caucasoid) | 99.05% | 0.00% | 99.05% |
| [Cuba Mixed](http://tools.iedb.org/population/result/#Cuba%20Mixed) | 0.00% | 85.48% | 85.48% |
| [Cuba Mulatto](http://tools.iedb.org/population/result/#Cuba%20Mulatto) | 98.74% | 0.00% | 98.74% |
| [Czech Republic](http://tools.iedb.org/population/result/#Czech%20Republic) | 99.08% | 86.21% | 99.87% |
| [Czech Republic Caucasoid](http://tools.iedb.org/population/result/#Czech%20Republic%20Caucasoid) | 99.08% | 88.76% | 99.90% |
| [Czech Republic Other](http://tools.iedb.org/population/result/#Czech%20Republic%20Other) | 0.00% | 64.14% | 64.14% |
| [Denmark](http://tools.iedb.org/population/result/#Denmark) | 0.00% | 88.98% | 88.98% |
| [Denmark Caucasoid](http://tools.iedb.org/population/result/#Denmark%20Caucasoid) | 0.00% | 88.98% | 88.98% |
| [East Africa](http://tools.iedb.org/population/result/#East%20Africa) | 90.78% | 68.30% | 97.08% |
| [East Asia](http://tools.iedb.org/population/result/#East%20Asia) | 98.18% | 81.82% | 99.67% |
| [Ecuador](http://tools.iedb.org/population/result/#Ecuador) | 77.35% | 52.17% | 89.16% |
| [Ecuador Amerindian](http://tools.iedb.org/population/result/#Ecuador%20Amerindian) | 77.35% | 52.17% | 89.16% |
| [England](http://tools.iedb.org/population/result/#England) | 99.93% | 93.48% | 100.00% |
| [England Caucasoid](http://tools.iedb.org/population/result/#England%20Caucasoid) | 99.93% | 93.48% | 100.00% |
| [Equatorial Guinea](http://tools.iedb.org/population/result/#Equatorial%20Guinea) | 0.00% | 47.58% | 47.58% |
| [Equatorial Guinea Black](http://tools.iedb.org/population/result/#Equatorial%20Guinea%20Black) | 0.00% | 47.58% | 47.58% |
| [Ethiopia](http://tools.iedb.org/population/result/#Ethiopia) | 0.00% | 83.00% | 83.00% |
| [Ethiopia Black](http://tools.iedb.org/population/result/#Ethiopia%20Black) | 0.00% | 83.00% | 83.00% |
| [Europe](http://tools.iedb.org/population/result/#Europe) | 99.68% | 85.83% | 99.96% |
| [Fiji](http://tools.iedb.org/population/result/#Fiji) | 0.00% | 79.87% | 79.87% |
| [Fiji Melanesian](http://tools.iedb.org/population/result/#Fiji%20Melanesian) | 0.00% | 79.87% | 79.87% |
| [Finland](http://tools.iedb.org/population/result/#Finland) | 99.99% | 51.14% | 100.00% |
| [Finland Caucasoid](http://tools.iedb.org/population/result/#Finland%20Caucasoid) | 99.99% | 51.14% | 100.00% |
| [France](http://tools.iedb.org/population/result/#France) | 99.80% | 88.54% | 99.98% |
| [France Caucasoid](http://tools.iedb.org/population/result/#France%20Caucasoid) | 99.80% | 88.54% | 99.98% |
| [Gabon](http://tools.iedb.org/population/result/#Gabon) | 0.00% | 41.78% | 41.78% |
| [Gabon Black](http://tools.iedb.org/population/result/#Gabon%20Black) | 0.00% | 41.78% | 41.78% |
| [Georgia](http://tools.iedb.org/population/result/#Georgia) | 98.32% | 75.05% | 99.58% |
| [Georgia Caucasoid](http://tools.iedb.org/population/result/#Georgia%20Caucasoid) | 98.94% | 75.05% | 99.74% |
| [Georgia Kurd](http://tools.iedb.org/population/result/#Georgia%20Kurd) | 98.19% | 0.00% | 98.19% |
| [Germany](http://tools.iedb.org/population/result/#Germany) | 99.93% | 91.14% | 99.99% |
| [Germany Caucasoid](http://tools.iedb.org/population/result/#Germany%20Caucasoid) | 99.93% | 91.14% | 99.99% |
| [Greece](http://tools.iedb.org/population/result/#Greece) | 0.00% | 66.92% | 66.92% |
| [Greece Caucasoid](http://tools.iedb.org/population/result/#Greece%20Caucasoid) | 0.00% | 66.92% | 66.92% |
| [Guatemala](http://tools.iedb.org/population/result/#Guatemala) | 7.76% | 49.16% | 53.11% |
| [Guatemala Amerindian](http://tools.iedb.org/population/result/#Guatemala%20Amerindian) | 7.76% | 49.16% | 53.11% |
| [Guinea-Bissau](http://tools.iedb.org/population/result/#Guinea-Bissau) | 96.39% | 71.16% | 98.96% |
| [Guinea-Bissau Black](http://tools.iedb.org/population/result/#Guinea-Bissau%20Black) | 96.39% | 71.16% | 98.96% |
| [Hong Kong](http://tools.iedb.org/population/result/#Hong%20Kong) | 96.05% | 0.00% | 96.05% |
| [Hong Kong Oriental](http://tools.iedb.org/population/result/#Hong%20Kong%20Oriental) | 96.05% | 0.00% | 96.05% |
| [India](http://tools.iedb.org/population/result/#India) | 89.41% | 74.99% | 97.35% |
| [India Asian](http://tools.iedb.org/population/result/#India%20Asian) | 89.41% | 74.99% | 97.35% |
| [Indonesia](http://tools.iedb.org/population/result/#Indonesia) | 86.79% | 47.84% | 93.11% |
| [Indonesia Austronesian](http://tools.iedb.org/population/result/#Indonesia%20Austronesian) | 86.79% | 47.84% | 93.11% |
| [Iran](http://tools.iedb.org/population/result/#Iran) | 97.50% | 64.22% | 99.11% |
| [Iran Kurd](http://tools.iedb.org/population/result/#Iran%20Kurd) | 0.00% | 55.78% | 55.78% |
| [Iran Persian](http://tools.iedb.org/population/result/#Iran%20Persian) | 97.50% | 65.72% | 99.14% |
| [Ireland Northern](http://tools.iedb.org/population/result/#Ireland%20Northern) | 99.94% | 94.65% | 100.00% |
| [Ireland Northern Caucasoid](http://tools.iedb.org/population/result/#Ireland%20Northern%20Caucasoid) | 99.94% | 94.65% | 100.00% |
| [Ireland South](http://tools.iedb.org/population/result/#Ireland%20South) | 99.93% | 93.15% | 100.00% |
| [Ireland South Caucasoid](http://tools.iedb.org/population/result/#Ireland%20South%20Caucasoid) | 99.93% | 93.15% | 100.00% |
| [Israel](http://tools.iedb.org/population/result/#Israel) | 89.39% | 68.79% | 96.69% |
| [Israel Arab](http://tools.iedb.org/population/result/#Israel%20Arab) | 94.42% | 67.51% | 98.19% |
| [Israel Jew](http://tools.iedb.org/population/result/#Israel%20Jew) | 93.75% | 69.65% | 98.10% |
| [Italy](http://tools.iedb.org/population/result/#Italy) | 99.03% | 85.90% | 99.86% |
| [Italy Caucasoid](http://tools.iedb.org/population/result/#Italy%20Caucasoid) | 99.03% | 85.90% | 99.86% |
| [Ivory Coast](http://tools.iedb.org/population/result/#Ivory%20Coast) | 67.75% | 0.00% | 67.75% |
| [Ivory Coast Black](http://tools.iedb.org/population/result/#Ivory%20Coast%20Black) | 67.75% | 0.00% | 67.75% |
| [Jamaica](http://tools.iedb.org/population/result/#Jamaica) | 0.00% | 27.41% | 27.41% |
| [Jamaica Black](http://tools.iedb.org/population/result/#Jamaica%20Black) | 0.00% | 27.41% | 27.41% |
| [Japan](http://tools.iedb.org/population/result/#Japan) | 98.63% | 74.83% | 99.66% |
| [Japan Oriental](http://tools.iedb.org/population/result/#Japan%20Oriental) | 98.63% | 74.83% | 99.66% |
| [Jordan](http://tools.iedb.org/population/result/#Jordan) | 90.83% | 52.88% | 95.68% |
| [Jordan Arab](http://tools.iedb.org/population/result/#Jordan%20Arab) | 90.83% | 52.88% | 95.68% |
| [Kenya](http://tools.iedb.org/population/result/#Kenya) | 89.56% | 0.00% | 89.56% |
| [Kenya Black](http://tools.iedb.org/population/result/#Kenya%20Black) | 89.56% | 0.00% | 89.56% |
| [Kiribati](http://tools.iedb.org/population/result/#Kiribati) | 0.00% | 10.89% | 10.89% |
| [Kiribati Micronesian](http://tools.iedb.org/population/result/#Kiribati%20Micronesian) | 0.00% | 10.89% | 10.89% |
| [Korea; South](http://tools.iedb.org/population/result/#Korea;%20South) | 98.21% | 85.32% | 99.74% |
| [Korea; South Oriental](http://tools.iedb.org/population/result/#Korea;%20South%20Oriental) | 98.21% | 85.32% | 99.74% |
| [Lebanon](http://tools.iedb.org/population/result/#Lebanon) | 0.00% | 70.46% | 70.46% |
| [Lebanon Arab](http://tools.iedb.org/population/result/#Lebanon%20Arab) | 0.00% | 70.46% | 70.46% |
| [Macedonia](http://tools.iedb.org/population/result/#Macedonia) | 26.72% | 66.53% | 75.47% |
| [Macedonia Caucasoid](http://tools.iedb.org/population/result/#Macedonia%20Caucasoid) | 26.72% | 66.53% | 75.47% |
| [Malaysia](http://tools.iedb.org/population/result/#Malaysia) | 81.38% | 57.99% | 92.18% |
| [Malaysia Austronesian](http://tools.iedb.org/population/result/#Malaysia%20Austronesian) | 63.18% | 55.38% | 83.57% |
| [Malaysia Oriental](http://tools.iedb.org/population/result/#Malaysia%20Oriental) | 87.82% | 70.35% | 96.39% |
| [Mali](http://tools.iedb.org/population/result/#Mali) | 96.02% | 0.00% | 96.02% |
| [Mali Black](http://tools.iedb.org/population/result/#Mali%20Black) | 96.02% | 0.00% | 96.02% |
| [Martinique](http://tools.iedb.org/population/result/#Martinique) | 22.56% | 74.51% | 80.26% |
| [Martinique Black](http://tools.iedb.org/population/result/#Martinique%20Black) | 22.56% | 74.51% | 80.26% |
| [Mexico](http://tools.iedb.org/population/result/#Mexico) | 97.97% | 55.04% | 99.09% |
| [Mexico Amerindian](http://tools.iedb.org/population/result/#Mexico%20Amerindian) | 99.87% | 42.59% | 99.93% |
| [Mexico Mestizo](http://tools.iedb.org/population/result/#Mexico%20Mestizo) | 98.13% | 68.51% | 99.41% |
| [Mongolia](http://tools.iedb.org/population/result/#Mongolia) | 95.31% | 81.85% | 99.15% |
| [Mongolia Oriental](http://tools.iedb.org/population/result/#Mongolia%20Oriental) | 95.31% | 81.85% | 99.15% |
| [Morocco](http://tools.iedb.org/population/result/#Morocco) | 98.63% | 83.44% | 99.77% |
| [Morocco Arab](http://tools.iedb.org/population/result/#Morocco%20Arab) | 99.32% | 85.07% | 99.90% |
| [Morocco Caucasoid](http://tools.iedb.org/population/result/#Morocco%20Caucasoid) | 97.94% | 79.75% | 99.58% |
| [Nauru](http://tools.iedb.org/population/result/#Nauru) | 0.00% | 38.66% | 38.66% |
| [Nauru Micronesian](http://tools.iedb.org/population/result/#Nauru%20Micronesian) | 0.00% | 38.66% | 38.66% |
| [Netherlands](http://tools.iedb.org/population/result/#Netherlands) | 0.00% | 83.44% | 83.44% |
| [Netherlands Caucasoid](http://tools.iedb.org/population/result/#Netherlands%20Caucasoid) | 0.00% | 83.44% | 83.44% |
| [New Caledonia](http://tools.iedb.org/population/result/#New%20Caledonia) | 98.18% | 81.41% | 99.66% |
| [New Caledonia Melanesian](http://tools.iedb.org/population/result/#New%20Caledonia%20Melanesian) | 98.18% | 81.41% | 99.66% |
| [New Zealand](http://tools.iedb.org/population/result/#New%20Zealand) | 0.00% | 84.46% | 84.46% |
| [New Zealand Polynesian](http://tools.iedb.org/population/result/#New%20Zealand%20Polynesian) | 0.00% | 84.46% | 84.46% |
| [Niue](http://tools.iedb.org/population/result/#Niue) | 0.00% | 77.82% | 77.82% |
| [Niue Polynesian](http://tools.iedb.org/population/result/#Niue%20Polynesian) | 0.00% | 77.82% | 77.82% |
| [North Africa](http://tools.iedb.org/population/result/#North%20Africa) | 96.03% | 75.06% | 99.01% |
| [North America](http://tools.iedb.org/population/result/#North%20America) | 99.06% | 87.89% | 99.89% |
| [Northeast Asia](http://tools.iedb.org/population/result/#Northeast%20Asia) | 94.70% | 59.99% | 97.88% |
| [Norway](http://tools.iedb.org/population/result/#Norway) | 0.00% | 94.71% | 94.71% |
| [Norway Caucasoid](http://tools.iedb.org/population/result/#Norway%20Caucasoid) | 0.00% | 94.71% | 94.71% |
| [Oceania](http://tools.iedb.org/population/result/#Oceania) | 94.71% | 59.87% | 97.88% |
| [Oman](http://tools.iedb.org/population/result/#Oman) | 99.69% | 0.00% | 99.69% |
| [Oman Arab](http://tools.iedb.org/population/result/#Oman%20Arab) | 99.69% | 0.00% | 99.69% |
| [Pakistan](http://tools.iedb.org/population/result/#Pakistan) | 97.09% | 1.18% | 97.13% |
| [Pakistan Asian](http://tools.iedb.org/population/result/#Pakistan%20Asian) | 96.75% | 1.45% | 96.79% |
| [Pakistan Mixed](http://tools.iedb.org/population/result/#Pakistan%20Mixed) | 97.73% | 0.00% | 97.73% |
| [Papua New Guinea](http://tools.iedb.org/population/result/#Papua%20New%20Guinea) | 97.92% | 69.15% | 99.36% |
| [Papua New Guinea Melanesian](http://tools.iedb.org/population/result/#Papua%20New%20Guinea%20Melanesian) | 97.92% | 69.15% | 99.36% |
| [Paraguay](http://tools.iedb.org/population/result/#Paraguay) | 0.00% | 4.90% | 4.90% |
| [Paraguay Amerindian](http://tools.iedb.org/population/result/#Paraguay%20Amerindian) | 0.00% | 4.90% | 4.90% |
| [Peru](http://tools.iedb.org/population/result/#Peru) | 99.99% | 49.87% | 100.00% |
| [Peru Amerindian](http://tools.iedb.org/population/result/#Peru%20Amerindian) | 99.99% | 49.87% | 100.00% |
| [Philippines](http://tools.iedb.org/population/result/#Philippines) | 94.98% | 28.56% | 96.41% |
| [Philippines Austronesian](http://tools.iedb.org/population/result/#Philippines%20Austronesian) | 94.98% | 28.56% | 96.41% |
| [Poland](http://tools.iedb.org/population/result/#Poland) | 99.77% | 84.46% | 99.96% |
| [Poland Caucasoid](http://tools.iedb.org/population/result/#Poland%20Caucasoid) | 99.77% | 84.46% | 99.96% |
| [Portugal](http://tools.iedb.org/population/result/#Portugal) | 98.72% | 78.00% | 99.72% |
| [Portugal Caucasoid](http://tools.iedb.org/population/result/#Portugal%20Caucasoid) | 98.72% | 78.00% | 99.72% |
| [Romania](http://tools.iedb.org/population/result/#Romania) | 99.67% | 0.00% | 99.67% |
| [Romania Caucasoid](http://tools.iedb.org/population/result/#Romania%20Caucasoid) | 99.67% | 0.00% | 99.67% |
| [Russia](http://tools.iedb.org/population/result/#Russia) | 99.27% | 77.62% | 99.84% |
| [Russia Caucasoid](http://tools.iedb.org/population/result/#Russia%20Caucasoid) | 3.96% | 88.52% | 88.97% |
| [Russia Mixed](http://tools.iedb.org/population/result/#Russia%20Mixed) | 5.05% | 0.00% | 5.05% |
| [Russia Other](http://tools.iedb.org/population/result/#Russia%20Other) | 99.98% | 85.01% | 100.00% |
| [Russia Siberian](http://tools.iedb.org/population/result/#Russia%20Siberian) | 99.43% | 78.83% | 99.88% |
| [Rwanda](http://tools.iedb.org/population/result/#Rwanda) | 24.87% | 62.79% | 72.05% |
| [Rwanda Black](http://tools.iedb.org/population/result/#Rwanda%20Black) | 24.87% | 62.79% | 72.05% |
| [Samoa](http://tools.iedb.org/population/result/#Samoa) | 0.00% | 80.86% | 80.86% |
| [Samoa Polynesian](http://tools.iedb.org/population/result/#Samoa%20Polynesian) | 0.00% | 80.86% | 80.86% |
| [Sao Tome and Principe](http://tools.iedb.org/population/result/#Sao%20Tome%20and%20Principe) | 97.02% | 66.50% | 99.00% |
| [Sao Tome and Principe Black](http://tools.iedb.org/population/result/#Sao%20Tome%20and%20Principe%20Black) | 97.02% | 66.50% | 99.00% |
| [Saudi Arabia](http://tools.iedb.org/population/result/#Saudi%20Arabia) | 98.26% | 80.14% | 99.65% |
| [Saudi Arabia Arab](http://tools.iedb.org/population/result/#Saudi%20Arabia%20Arab) | 98.26% | 80.14% | 99.65% |
| [Scotland](http://tools.iedb.org/population/result/#Scotland) | 65.34% | 90.82% | 96.82% |
| [Scotland Caucasoid](http://tools.iedb.org/population/result/#Scotland%20Caucasoid) | 65.34% | 90.82% | 96.82% |
| [Senegal](http://tools.iedb.org/population/result/#Senegal) | 95.58% | 30.28% | 96.92% |
| [Senegal Black](http://tools.iedb.org/population/result/#Senegal%20Black) | 95.58% | 30.28% | 96.92% |
| [Serbia](http://tools.iedb.org/population/result/#Serbia) | 73.37% | 0.00% | 73.37% |
| [Serbia Caucasoid](http://tools.iedb.org/population/result/#Serbia%20Caucasoid) | 73.37% | 0.00% | 73.37% |
| [Singapore](http://tools.iedb.org/population/result/#Singapore) | 92.66% | 65.78% | 97.49% |
| [Singapore Austronesian](http://tools.iedb.org/population/result/#Singapore%20Austronesian) | 90.55% | 65.78% | 96.77% |
| [Singapore Oriental](http://tools.iedb.org/population/result/#Singapore%20Oriental) | 94.81% | 0.00% | 94.81% |
| [Slovakia](http://tools.iedb.org/population/result/#Slovakia) | 0.00% | 18.28% | 18.28% |
| [Slovakia Caucasoid](http://tools.iedb.org/population/result/#Slovakia%20Caucasoid) | 0.00% | 18.28% | 18.28% |
| [Slovenia](http://tools.iedb.org/population/result/#Slovenia) | 0.00% | 84.85% | 84.85% |
| [Slovenia Caucasoid](http://tools.iedb.org/population/result/#Slovenia%20Caucasoid) | 0.00% | 84.85% | 84.85% |
| [South Africa](http://tools.iedb.org/population/result/#South%20Africa) | 93.03% | 32.10% | 95.27% |
| [South Africa Black](http://tools.iedb.org/population/result/#South%20Africa%20Black) | 91.96% | 32.10% | 94.54% |
| [South Africa Other](http://tools.iedb.org/population/result/#South%20Africa%20Other) | 97.61% | 0.00% | 97.61% |
| [South America](http://tools.iedb.org/population/result/#South%20America) | 88.30% | 58.59% | 95.15% |
| [South Asia](http://tools.iedb.org/population/result/#South%20Asia) | 94.73% | 75.38% | 98.70% |
| [Southeast Asia](http://tools.iedb.org/population/result/#Southeast%20Asia) | 94.56% | 56.98% | 97.66% |
| [Southwest Asia](http://tools.iedb.org/population/result/#Southwest%20Asia) | 92.50% | 43.93% | 95.79% |
| [Spain](http://tools.iedb.org/population/result/#Spain) | 87.52% | 80.51% | 97.57% |
| [Spain Caucasoid](http://tools.iedb.org/population/result/#Spain%20Caucasoid) | 87.52% | 80.84% | 97.61% |
| [Spain Other](http://tools.iedb.org/population/result/#Spain%20Other) | 0.00% | 6.30% | 6.30% |
| [Sri Lanka](http://tools.iedb.org/population/result/#Sri%20Lanka) | 52.39% | 0.00% | 52.39% |
| [Sri Lanka Asian](http://tools.iedb.org/population/result/#Sri%20Lanka%20Asian) | 52.39% | 0.00% | 52.39% |
| [Sudan](http://tools.iedb.org/population/result/#Sudan) | 93.78% | 60.56% | 97.55% |
| [Sudan Arab](http://tools.iedb.org/population/result/#Sudan%20Arab) | 70.21% | 0.00% | 70.21% |
| [Sudan Black](http://tools.iedb.org/population/result/#Sudan%20Black) | 2.19% | 0.00% | 2.19% |
| [Sudan Mixed](http://tools.iedb.org/population/result/#Sudan%20Mixed) | 94.39% | 60.56% | 97.79% |
| [Sweden](http://tools.iedb.org/population/result/#Sweden) | 99.99% | 88.07% | 100.00% |
| [Sweden Caucasoid](http://tools.iedb.org/population/result/#Sweden%20Caucasoid) | 99.99% | 88.07% | 100.00% |
| [Taiwan](http://tools.iedb.org/population/result/#Taiwan) | 97.77% | 67.88% | 99.29% |
| [Taiwan Oriental](http://tools.iedb.org/population/result/#Taiwan%20Oriental) | 97.77% | 67.88% | 99.29% |
| [Thailand](http://tools.iedb.org/population/result/#Thailand) | 91.21% | 63.90% | 96.83% |
| [Thailand Oriental](http://tools.iedb.org/population/result/#Thailand%20Oriental) | 91.21% | 63.90% | 96.83% |
| [Tokelau](http://tools.iedb.org/population/result/#Tokelau) | 0.00% | 55.11% | 55.11% |
| [Tokelau Polynesian](http://tools.iedb.org/population/result/#Tokelau%20Polynesian) | 0.00% | 55.11% | 55.11% |
| [Tonga](http://tools.iedb.org/population/result/#Tonga) | 0.00% | 71.91% | 71.91% |
| [Tonga Polynesian](http://tools.iedb.org/population/result/#Tonga%20Polynesian) | 0.00% | 71.91% | 71.91% |
| [Tunisia](http://tools.iedb.org/population/result/#Tunisia) | 97.52% | 74.26% | 99.36% |
| [Tunisia Arab](http://tools.iedb.org/population/result/#Tunisia%20Arab) | 97.52% | 74.97% | 99.38% |
| [Tunisia Berber](http://tools.iedb.org/population/result/#Tunisia%20Berber) | 0.00% | 74.47% | 74.47% |
| [Turkey](http://tools.iedb.org/population/result/#Turkey) | 44.80% | 76.19% | 86.85% |
| [Turkey Caucasoid](http://tools.iedb.org/population/result/#Turkey%20Caucasoid) | 44.80% | 76.19% | 86.85% |
| [Uganda](http://tools.iedb.org/population/result/#Uganda) | 94.87% | 0.00% | 94.87% |
| [Uganda Black](http://tools.iedb.org/population/result/#Uganda%20Black) | 94.87% | 0.00% | 94.87% |
| [Ukraine](http://tools.iedb.org/population/result/#Ukraine) | 0.00% | 50.64% | 50.64% |
| [Ukraine Caucasoid](http://tools.iedb.org/population/result/#Ukraine%20Caucasoid) | 0.00% | 50.64% | 50.64% |
| [United Arab Emirates](http://tools.iedb.org/population/result/#United%20Arab%20Emirates) | 3.37% | 32.92% | 35.19% |
| [United Arab Emirates Arab](http://tools.iedb.org/population/result/#United%20Arab%20Emirates%20Arab) | 3.37% | 32.92% | 35.19% |
| [United States](http://tools.iedb.org/population/result/#United%20States) | 99.10% | 88.10% | 99.89% |
| [United States Amerindian](http://tools.iedb.org/population/result/#United%20States%20Amerindian) | 99.67% | 42.79% | 99.81% |
| [United States Asian](http://tools.iedb.org/population/result/#United%20States%20Asian) | 97.46% | 78.84% | 99.46% |
| [United States Austronesian](http://tools.iedb.org/population/result/#United%20States%20Austronesian) | 0.00% | 58.09% | 58.09% |
| [United States Black](http://tools.iedb.org/population/result/#United%20States%20Black) | 97.11% | 71.50% | 99.18% |
| [United States Caucasoid](http://tools.iedb.org/population/result/#United%20States%20Caucasoid) | 99.83% | 90.15% | 99.98% |
| [United States Hispanic](http://tools.iedb.org/population/result/#United%20States%20Hispanic) | 98.92% | 72.95% | 99.71% |
| [United States Mestizo](http://tools.iedb.org/population/result/#United%20States%20Mestizo) | 99.23% | 72.23% | 99.79% |
| [United States Polynesian](http://tools.iedb.org/population/result/#United%20States%20Polynesian) | 99.57% | 73.18% | 99.88% |
| [Venezuela](http://tools.iedb.org/population/result/#Venezuela) | 90.04% | 3.01% | 90.34% |
| [Venezuela Amerindian](http://tools.iedb.org/population/result/#Venezuela%20Amerindian) | 90.07% | 0.00% | 90.07% |
| [Venezuela Caucasoid](http://tools.iedb.org/population/result/#Venezuela%20Caucasoid) | 11.45% | 0.00% | 11.45% |
| [Venezuela Mestizo](http://tools.iedb.org/population/result/#Venezuela%20Mestizo) | 9.75% | 0.00% | 9.75% |
| [Venezuela Mixed](http://tools.iedb.org/population/result/#Venezuela%20Mixed) | 0.00% | 3.17% | 3.17% |
| [Vietnam](http://tools.iedb.org/population/result/#Vietnam) | 91.82% | 54.44% | 96.27% |
| [Vietnam Oriental](http://tools.iedb.org/population/result/#Vietnam%20Oriental) | 91.82% | 54.44% | 96.27% |
| [Wales](http://tools.iedb.org/population/result/#Wales) | 1.00% | 0.00% | 1.00% |
| [Wales Caucasoid](http://tools.iedb.org/population/result/#Wales%20Caucasoid) | 1.00% | 0.00% | 1.00% |
| [West Africa](http://tools.iedb.org/population/result/#West%20Africa) | 95.49% | 65.23% | 98.43% |
| [West Indies](http://tools.iedb.org/population/result/#West%20Indies) | 98.98% | 69.22% | 99.69% |
| [World](http://tools.iedb.org/population/result/#World) | 98.55% | 81.81% | 99.74% |
| [Zambia](http://tools.iedb.org/population/result/#Zambia) | 98.10% | 0.00% | 98.10% |
| [Zambia Black](http://tools.iedb.org/population/result/#Zambia%20Black) | 98.10% | 0.00% | 98.10% |
| [Zimbabwe](http://tools.iedb.org/population/result/#Zimbabwe) | 93.79% | 68.30% | 98.03% |
| [Zimbabwe Black](http://tools.iedb.org/population/result/#Zimbabwe%20Black) | 93.79% | 68.30% | 98.03% |

**Table S-2. Top 10 model and its RMSD, MolProbity, Clash score, Poor rotamers, Rama favored and predicted GALAXY energy scouring.**

| Model | RMSD | MolProbity | Clash score | Poor rotamers | Rama favored | GALAXY energy |
| --- | --- | --- | --- | --- | --- | --- |
| Initial | 0.000 | 3.646 | 105.0 | 6.7 | 90.5 | 26423.14 |
| MODEL 1 | 0.967 | 1.142 | 1.2 | 0.5 | 95.7 | -4466.32 |
| MODEL 2 | 1.047 | 1.270 | 1.5 | 0.0 | 94.4 | -4458.51 |
| MODEL 3 | 1.008 | 1.270 | 1.5 | 0.5 | 94.4 | -4457.28 |
| MODEL 4 | 1.872 | 1.381 | 1.5 | 0.0 | 91.8 | -4452.32 |
| MODEL 5 | 0.994 | 1.230 | 2.0 | 0.0 | 96.1 | -4442.66 |
| MODEL 6 | 1.206 | 1.313 | 1.5 | 0.0 | 93.5 | -4437.67 |
| MODEL 7 | 1.995 | 1.150 | 1.0 | 0.5 | 94.8 | -4435.53 |
| MODEL 8 | 1.507 | 1.292 | 1.5 | 0.5 | 94.0 | -4435.20 |
| MODEL 9 | 0.919 | 1.357 | 2.2 | 0.0 | 94.8 | -4432.05 |
| MODEL 10 | 0.904 | 1.265 | 2.0 | 0.0 | 95.7 | -4431.51 |

**Table S-3. Docking score of vaccine and TLR-2**

| **Cluster** | **Members** | **Representative** | **Weighted Score** |
| --- | --- | --- | --- |
| **1** | 62 | Center | -1023.4 |
|  |  | Lowest Energy | -1206.1 |
| **2** | 55 | Center | -938.1 |
|  |  | Lowest Energy | -1099.3 |
| **3** | 51 | Center | -936.8 |
|  |  | Lowest Energy | -1132.0 |
| **4** | 47 | Center | -1506.8 |
|  |  | Lowest Energy | -1506.8 |
| **5** | 44 | Center | -955.0 |
|  |  | Lowest Energy | -1135.4 |
| **6** | 39 | Center | -928.2 |
|  |  | Lowest Energy | -1087.4 |
| **7** | 37 | Center | -988.9 |
|  |  | Lowest Energy | -1184.5 |
| **8** | 35 | Center | -1013.7 |
|  |  | Lowest Energy | -1013.7 |
| **9** | 32 | Center | -948.6 |
|  |  | Lowest Energy | -1122.1 |
| **10** | 27 | Center | -976.0 |
|  |  | Lowest Energy | -1115.7 |

**Table S-4. Docking score of vaccine and TLR-4**

| **Cluster** | **Members** | **Representative** | **Weighted Score** |
| --- | --- | --- | --- |
| **1** | 74 | Center | -1191.5 |
|  |  | Lowest Energy | -1191.5 |
| **2** | 62 | Center | -1243.6 |
|  |  | Lowest Energy | -1314.9 |
| **3** | 55 | Center | -967.2 |
|  |  | Lowest Energy | -1141.1 |
| **4** | 43 | Center | -1073.9 |
|  |  | Lowest Energy | -1141.9 |
| **5** | 38 | Center | -1122.3 |
|  |  | Lowest Energy | -1122.3 |
| **6** | 32 | Center | -984.5 |
|  |  | Lowest Energy | -1146.1 |
| **7** | 31 | Center | -1080.4 |
|  |  | Lowest Energy | -1150.9 |
| **8** | 31 | Center | -969.0 |
|  |  | Lowest Energy | -1059.7 |
| **9** | 28 | Center | -1077.3 |
|  |  | Lowest Energy | -1175.7 |
| **10** | 27 | Center | -1045.0 |
|  |  | Lowest Energy | -1101.0 |
